# Breast Cancer and the Other Non-Coding RNAs

**DOI:** 10.3390/ijms22063280

**Published:** 2021-03-23

**Authors:** Dana Dvorská, Dušan Braný, Marcela Ňachajová, Erika Halašová, Zuzana Danková

**Affiliations:** 1 Biomedical Center Martin, Jessenius Faculty of Medicine in Martin, Comenius University in Bratislava, 036 01 Martin, Slovakia; dana.dvorska@uniba.sk (D.D.); erika.halasova@uniba.sk (E.H.); zuzana.dankova@uniba.sk (Z.D.); 2 Department of Obstetrics and Gynaecology, Martin University Hospital, Jessenius Faculty of Medicine, Comenius University in Bratislava, 036 01 Martin, Slovakia; marcela.nachajova@uniba.sk

**Keywords:** non-coding RNA, long non-coding RNA, Piwi-interacting RNA, breast cancer, RNA interference, small nucleolar RNA, small nuclear RNA

## Abstract

Breast cancer is very heterogenous and the most common gynaecological cancer, with various factors affecting its development. While its impact on human lives and national health budgets is still rising in almost all global areas, many molecular mechanisms affecting its onset and development remain unclear. Conventional treatments still prove inadequate in some aspects, and appropriate molecular therapeutic targets are required for improved outcomes. Recent scientific interest has therefore focused on the non-coding RNAs roles in tumour development and their potential as therapeutic targets. These RNAs comprise the majority of the human transcript and their broad action mechanisms range from gene silencing to chromatin remodelling. Many non-coding RNAs also have altered expression in breast cancer cell lines and tissues, and this is often connected with increased proliferation, a degraded extracellular environment, and higher endothelial to mesenchymal transition. Herein, we summarise the known abnormalities in the function and expression of long non-coding RNAs, Piwi interacting RNAs, small nucleolar RNAs and small nuclear RNAs in breast cancer, and how these abnormalities affect the development of this deadly disease. Finally, the use of RNA interference to suppress breast cancer growth is summarised.

## 1. Introduction

Breast cancer (BC) is the most common gynaecological cancer, and it was identified in 2018 as the main cause of female cancer death in 11 of the 20 major global regions [1]. There was recorded annual increase to 3.1% between 1980 and 2015, and the incidence continues to rise [2]. While this incidence is 92 in 100,000 in North America [3] and 144 in 100,000 in European Union countries [4], BC is diagnosed in Asian countries approximately 20 years earlier, at an average 40–50 years compared to 50–70 in European countries. Africa and Central Asia, however, record the lowest BC incidence, but the incidence there is most likely influenced by insufficient screening and diagnostic programmes [5,6]. In addition, BC is diagnosed in the earlier stages in the more developed countries and although it is more common there, the overall mortality is lower. Finally, the highest BC mortality is recorded in sub-Saharan Africa [7], where women of African origin are more prone to rapid metastasising BC with lower survival rate [8].

BC comprises variable tumours with different histological, clinical and molecular-biological manifestations [9]. The basic histological division of BC tumours is into invasive and pre-invasive types. The latter has two distinct entities, lobular carcinoma in-situ (LCIS) and ductal carcinoma in-situ (DCIS). The risk of LCIS progression is very low, and these lesions are considered more a risk factor than an invasive lesion precursor. This type can be bilateral, but it does not usually distort lobular architecture. In contrast, DCIS are unilateral and can spread through ducts, thus disturbing ductal architecture. This type comprises more than 80% of all pre-invasive lesions and it can progress to invasive forms [7,9,10].

The invasive BC subtypes spread into surrounding breast tissue, and the most frequent of these are invasive carcinoma of no special type (NST; formerly known as invasive ductal carcinoma) and invasive lobular carcinoma. NST accounts for 70–75% of all BCs, followed by lobular carcinomas with 10–15% and the remaining BC tumours form 17 rare histological subtypes [9,11]. Finally, only up to 10% of all diagnosed BC are non-invasive, and 20–53% of women with DCIS develop invasive carcinoma within 10 years [12].

Initial BC molecular classification was introduced by Perou et Sørlie [13] at the beginning of the millennium, and this enabled BC tumour classification into Luminal, HER2 positive, basal and ‘normal-like’ subtypes through differences in gene expression pattern. Perou and Sørlie’s pilot study inspired further research in molecular BC classification and several variations of molecular classification based on gene expression or mutations in particular genes have been created. However, molecular classification is still developing and cannot be considered established [14,15,16,17,18,19]. Current molecular classification is typically based on PAM50, which is a 50-gene expression signature that classifies BC in five molecular intrinsic subtypes: Luminal A, Luminal B, HER2-enriched, Basal-like, and Normal-like. Moreover, additional subtypes have been identified, including claudin-low, molecular apocrine and interferon-rich subtypes [14,15,16,17,18,19]. However, there is still no consensus whether to consider these “additional” subtypes analogous to the intrinsic subtypes, or if tumours carry these phenotypes only in addition to their intrinsic subtype [10,16].

In summary, although molecular classifications provide important characteristics of particular tumour types and can be beneficial in predicting tumour progression, molecular classifications are often too methodologically and financially demanding for daily clinical use [14,15,16,17,18,19]. These problems mean that current clinical practice typically uses a surrogate classification of five subtypes based on histology and immunohistochemistry This particularly involves assessing the expression of key proteins: oestrogen receptor (ER), progesterone receptor (PR), human epidermal growth factor receptor 2 (HER2) and the proliferation marker Ki67. These subtypes are: Luminal A-like, Luminal B-like HER2-, Luminal B-like Her2+, HER2-enriched, and Triple negative (TNBC) [9,10,20].

(1). Luminal A-like is the most common subtype, accounting for 60–70% of all BC tumours. These tumours have the best prognosis and overall survival rates. They are low grade tumours, ER and PR positive and HER2 and Ki67 negative (ER+; PR+; HER2−; Ki67− (<14%)).

(2). Luminal B-like HER2- is the second most common subtype, comprising almost 10–20% of BC tumours. These have intermediate prognosis, and although their ER and PR positivity is lower than Luminal A, they are still classified as ER+; PR+; HER2- and Ki67+ (>14%).

(3). Luminal B-like HER2+ have similar characteristics to the luminal-B like subtype, but are also positive for HER2. Their prognosis is worse than luminal B-like and often have higher grade.

(4). HER2-enriched are typically (ER−; PR−; HER2+, Ki67+). Although these tumours are considered aggressive, they respond reasonably well to therapy and their prognosis therefore remains intermediate. Together with the Luminal B -like HER2+ subtype they cover 15–20% of all BC tumours.

(5). TNBC accounts for approximately 15% of BC tumours and they are typically (ER-; PR-; HER2-, Ki67+). These are high grade with poor prognoses and positive for basal-markers with high Ki67 proliferation index.

The method of treatment for these BC tumours is directly related to the specific subtype [9,21].

(1). Endocrine therapy is prescribed by default for luminal subtypes if there is minimal risk of recurrence. In greater risk, the treatment of choice is neoadjuvant or adjuvant chemotherapy with docetaxel and cyclophosphamide if the tumour burden is low (pN0-1), and anthracycline-taxane sequence is preferred in higher tumour burden (pN2-3).

(2). Adjuvant chemotherapy with paclitaxel and trastuzumab is usually applied for 1 year when HER2+ tumours are pT1/pN0. If these tumours are ≥T2, or spread to lymph nodes, chemotherapy is preferred in neoadjuvant settings with anthracycline-taxane combined with dual HER2 blockade (trastuzumab and petruzumab). Further treatment then depends on complete pathological response, and treatment continues with Anti-HER2 therapy for one year when response is positive and T-DM1 is applied when it is negative.

(3). TNBC is considered the most aggressive subtype, and initial neoadjuvant chemotherapy with anthracycline-taxane sequence is preferred (platinum agent may be added). Adjuvant chemotherapy with capecitabine is then applied in the absence of complete pathological response.

However, this is only a generalised algorithm for treatment of early BC and the preference for adjuvant or non-adjuvant therapy and the use of particular pharmaceuticals depends on the precise situation, including menopausal status, concurrent treatments and other clinical factors [9,21].

The human genome contains 3 × 10^9^ base pairs, and the DNA in all chromosomes in the cell measures 2 metres when extended. While approximately 75% of this genetic information is further transcribed [22,23], only 1.5–2% of the total transcript is mRNA and this is further translated into amino acid sequence. The majority of the transcript comprises non-coding RNAs (ncRNA) which directly or indirectly regulate mRNA expression and the derived protein products. It is presumed that abnormal expression levels of the many genes involved in BC development are influenced by ncRNA activity [24,25]. Therefore, knowledge of particular ncRNA characteristics can help understanding complex BC cell mechanisms and improve BC subtype diagnosis and medical treatment.

The non-coding RNAs are divided into two main groups, house-keeping ncRNAs and regulatory ncRNAs. The house keeping ncRNAs are usually constitutively expressed under physiological conditions and include the following RNAs: transfer RNAs (tRNA), ribosomal RNAs (rRNA), small nuclear RNAs (snRNA), small nucleolar RNAs (snoRNA) and telomerase RNA (TERC). In contrast, the regulatory ncRNAs are expressed in a more cell-and-tissue-specific manner and often in response to environmental factors. These can regulate gene expression at the epigenetic, transcriptional, post-transcriptional, translational, and post-translational levels [26,27]. Regulatory non-coding RNAs are divided into short non-coding RNAs less than 200 bp and long non-coding RNAs (lncRNAs) with over 200 bp. The short ncRNAs are further sub-divided into microRNAs (miRNAs), short interfering RNAs (siRNAs) and Piwi-interacting RNAs (piRNAs) [22,23,28,29]. The majority of analysis and research has focused on miRNA rather than other ncRNAs, although the miRNAs constitute only a small percentage of the total transcript [24,25], This has inspired a summary of the effect of the more abundant, but less understood, non-coding RNAs on the development and progression of BC. This especially includes a description of the function lncRNAs, piRNAs, snRNAs and snoRNAs. Finally, the possibilities of inhibiting tumours by RNA interference (RNAi) are discussed.

## 2. microRNAs

**Figure 1:**
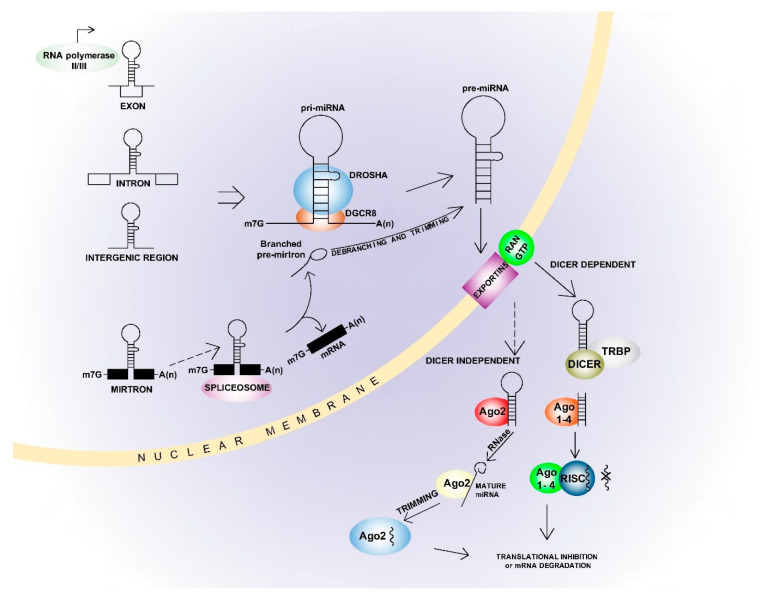
Canonical and non-canonical miRNA biogenesis pathways. miRNAs are usually transcribed by polymerase II; but some are also transcribed by polymerase III as in those miRNAs from clusters spread within Alu repeats. The primary transcript (pri-miRNA) in canonical biogenesis has typical loop structure after transcription and it can it be hundreds of base pairs long. The pri-miRNA is recognised by DGCR8 protein and this combines with Drosha enzyme to form a micro-processor complex which removes the miRNA tails and cuts pri-miRNA into smaller precursor miRNA (pre-miRNA). Exportin-5 transports this to the cytoplasm through nucleopores and the pre-miRNA is recognised there by the large Dicer RNAse protein. Dicer cleaves the stem loop and forms the mature double-stranded miRNA molecule. This is then loaded into the Argonaute protein family, the passenger strand is degraded and the ‘miRNA-induced silencing complex (miRISC) is formed. The miRISC then bouns to its target mRNA sequence, usually at the mRNA 3′UTR region. This miRISC can inactivate mRNA by direct cleavage, or physically prevent ribosome sub-unit binding.This figure also depicts two non-canonical miRNA biogenesis pathways; (1) ‘mirtron’ miRNAs are produced from introns during mRNA splicing, and this biogenesis is Drosha independent. Branched pre-mirtrons are formed after splicing, de-branched by lariat debranching enzyme (Ldbr), enzymatically trimmed and folded into pre-miRNA hairpins. (2) in Dicer independent biogenesis, the miRNAs are loaded directly into Ago2 protein which cleaves target strands in the middle of its 3′arm, and mature miRNA is then generated by poly(A)-specific ribonuclease’(PARN) trimming. miR-451 is the one known representative of Dicer independent biogenesis, and this is the most abundant miRNA in erythrocytes.

Although the main goal of this article is to summarise the effects of ncRNAs other than miRNAs on BC development, miRNAs are repeatedly discussed in the following text, especially in relation to their interaction with lncRNAs. The basic characteristics and mechanisms of miRNAs are therefore included herein. miRNAs range from 21 to 25 nt in length, but are most commonly 22 nt [30], and miRNA genes are mostly transcribed from both intragenic and intergenic regions by polymerase II. They are less often transcribed by polymerase III, and then especially from miRNA coding sequences spread among Alu repeats [31]. In addition, miRNAs from the same family are often co-transcribed in “clusters” [32]. miRNAs from the same family have a similar seed sequence, comprising nucleotides 2–8 from the 5′end. These sequences are essential for binding to mRNA [33]. Approximately 1900 pre-miRNAs and over 2600 mature mRNAs have been described in the human genome [30,34]. The miRNA synthesis pathways can be canonical or non-canonical (Figure 1) [30,31]. In the canonical pathway, miRNA genes are initially transcribed into the primary pri-miRNAs and then processed into the precursor pre-miRNAs. This process is mediated by a “micro-processor complex” comprising DGCR8, ribonuclease III and Drosha [35]. The pre-miRNAs are then exported to the cytoplasm by Exportin 5 and processed to mature miRNA’s by the Dicer complex [30,31]. Double-stranded miRNAs are separated into guide strand and passenger strand, depending on orientation and sequence stability. Here, the strand with lower thermodynamic stability at the 5′ end usually features as the guide strand for incorporation into the Argonaute protein while the passenger strand is degraded [30,31]. Non-canonical biogenesis varies depending on the protein complexes involved, and is most often Drosha/DGCR-8 independent or Dicer independent [30,31]. While the miRNAs most often interact with the mRNA 3′UTR region, they can also interact with the 5′UTR region or directly with the gene promoter region [36]. These interactions primarily result in repression of target gene expression, but some miRNAs also enhance gene expression [30,31]. This is usually seen in ‘starved’ cells, or under other non-physiological conditions [37,38]. In addition, one miRNA can inhibit several genes and one gene can be affected by multiple miRNAs [30,31]. This can result in complete cell signalling regulated by one or only a few miRNAs, and the miRNAs can have either a tumour-suppressive or oncogenic effect [39].

## 3. Long Non-Coding RNAs

lncRNAs have a greater than 200 bp sequence [26] and were initially considered redundant sequences following gene transcription. These lncRNAs undergo splicing and removal of intron-like sequences, similar to genes encoding for proteins. It is also currently estimated that over 270,000 lncRNAs are present in the human genome, although many have very low copy number [40]. The lncRNAs are therefore very abundant and heterogeneous, and their sequences can be located close to the genes, in the space between genes or even overlapping them. Their binding is especially variable: in the 3′-5′direction, the 5′-3′direction, bi-directional or binding to intergenic regions, intron regions, and enhancer sequences [41].

**Figure 2:**
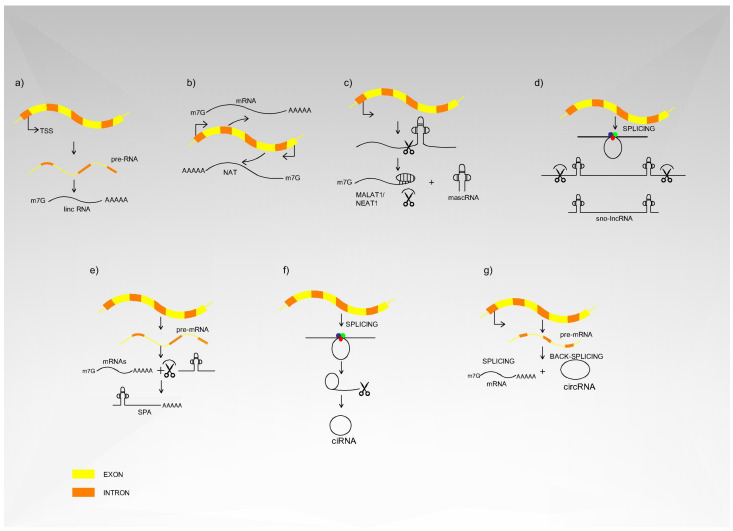
Variability in Long-noncoding RNA biogenesis. (**a**) Long intergenic non-coding RNAs (lincRNAs) are transcribed by Polymerase II from regions between two protein coding genes, and they are usually capped, polyadenylated and spliced as in mRNA, but some undergo only terminal cleavage and premature termination. (**b**) Natural antisense transcripts (NAT) are synthesised by RNA polymerase II from the antisense strand of the protein coding gene. There are three NAT forms – Complete, Intron-overlapped and Exon-overlaped. (**c**) MALAT1/NEAT1 is cleaved by RNAseP after transcription. The U-A-U structure stabilises its 3′ end and inhibits further cleavage and mascRNA 3′-end products with unknown function are also created. (**d**) sno lncRNAs are products of intron excision. The snoRNP complex is formed on both ends, and this protects sequence from further degradation. sno lncRNAs lack both capping and polyadenylation. (**e**) SPA lncRNAs have snoRNP at their 5′ends and 3′-ends and are polyadenylated. They originate as a product of read-through transcription, and this is followed by multistep 5′end trimming and 3′end processing. (**f**) Circular intronic RNAs are products of excision of intron with consensus sequence (5′splice site is GU rich and branchpoint site is C rich), 3′ end is usually trimmed and debranched. (**g**) Finally, the circular RNAs (circRNAs) are products of circular back-slicing of the pre-mRNA exons. Edited from [26] with permission.

The lncRNAs division is as follows [26]:

(a) mRNA-like transgenic transcripts, also known as lincRNAs. These are further spliced, capped and poly-adenylated

(b) Natural antisense transcripts (NAT) of protein coding genes

(c) primary RNA polymerase II transcript-derived unconventional lncRNAs. This group can be divided into the following subgroups based on the types of stabilisation and modifications they undergo:

(c1) MALAT and NEAT1 lncRNAs, processed by RNase P and stabilized by U-A-U triple helix structures at their 3′ ends; (c2) snoRNA-ended lncRNAs mainly derived from excised introns; (c3) 5′ snoRNA-ended and 3′-polyadenylated lncRNAs (SPA); (c4) Circular intronic RNAs (ciRNA) derived from excised introns; (c5) Circular RNAs (circRNA) produced by circular back-splicing of pre-mRNA exons. This division is depicted in Figure 2.

The lncRNAs also vary in their final effect. For example, they are preferentially located in the nucleus and can form a complex there with the HNRPNK nuclear matrix protein and participate in nuclear organisation [26,42,43]. Xist lncRNA is involved in inactivating one of the X chromosomes [44] and C0T-1 lncRNA affects chromosome decondensation [45].

lncRNAs also act on chromatin remodelling by variable cis and trans mechanisms. Recruiting the PRC2 polycomb repressive complex is common, and this progresses to deposition of histone H3 lysine 27 trimethylation (H3K27me3) [46]. Moreover, there is antagonistic effect of the MHRT (Myosin Heavy Chain Associated RNA Transcripts) cluster lncRNAs on the Brg1 catalytic subunit of the BAF chromatin remodelling complex [47]. lncRNAs can interact with chromatin remodelling complexes, primarily the SWI/SNF complex [48] and they can modify histones by altering their methylation [49] and acetylation [50], or affect DNA methylation levels [51,52]. Furthermore, they can either enhance or inhibit gene transcription as cis or trans regulators [53]. The lncRNAs are also more tissue specific than miRNAs and although some conservation is observed in the formation of secondary and tertiary structures in closely related species, individual species’ evolutionary conservation is generally low [54].

lncRNAs often function as competing endogenous RNAs (ceRNA) [55]. These lncRNAs contain complementary binding sites to miRNA called microRNA response elements (MRE).This results in miRNA binding to this lncRNA instead of binding to the target mRNA sequence, and the latter is therefore not repressed. In addition, this effect can also be achieved with artificially prepared constructs called “miRNA sponges” [40,55]. These are often circular and contain variable numbers of copies of desired MREs. Their binding sites are usually specific for seed region and this enables blocking of entire miRNA families. Finally, some lncRNAs abundant in BC are precursors for various miRNAs [26].

### 3.1. Long Non-Coding RNAs and BC

Description of H19, TINCR, MALAT and NEAT1 DANCR lncRNAs follows, because their abnormal expression is associated with BC development and metastasis. Additional lncRNAs reported to be abnormally expressed in at least one study and also lncRNAs associated with TNBC development are then summarised.

#### 3.1.1. H19 lncRNA

The association of dysregulated lncRNA H19 (H19) expression and BC development has been described [56,57]. Here, the highly expressed H19 is apparent in over 70% of BC tumours, and this includes ER+ and ER-, and HER2+ and HER2- [56,57,58]. Several typical mutational polymorphisms are also associated with higher expression of this lncRNA in BC [58,59]. H19 knockout can then suppress the Akt pathway, induce apoptosis, and restore paclitaxel sensitivity [56,57]. EZF1 also binds to the H19 promoter region, activates its expression [60] and promotes cell cycle progression. EZF1 is over-expressed in BC tumours [60].

In addition, lncRNA-H19 creates a precursor for miR-675 whose abnormal expression is demonstrably associated with BC development [56,61] and it also acts as a ceRNA in BC cells and interacts with miR-200b/c and let-7b. This increases the expression of *GIT2* and *CYTH3* genes, which are important contributors to rapid epithelial-to-mesenchymal transition (EMT) [62]. In addition, the 200b/c and let-7b miRNAs are often abnormally expressed in metastatic BC tumours [63]. The lncRNA-H19 can bind to miR-152 which targets DNA methyltransferase 1 (DNMT1), and this provides a further mechanism for altering BC methylation levels [64]. Furthermore, the lncRNA-H19 binding to miR-9-5p depletes the latter’s levels and increases the expression of the important *STAT3* signal transducer gene in BC [65].

Finally, lncRNA-H19 interacts with the Myc family of proto-oncogenes and these are among the most dysregulated transcription factors in various tumorous cells, including BC cells [56]. c-Myc affects histone acetylation and H19 transcription initiation [66], and n-Myc regulates DNA methyltransferase 3α2, thus altering H19 locus methylation status and expression [67].

#### 3.1.2. TINCR lncRNA

TINCR lncRNA (TINCR) affects the formation of primary BC tumours, and subsequent metastasis was discovered in 2018 [68]. The qPCR methodology in that study of 24 patients determined higher TINCR BC expression than in non-BC participants. In addition, higher TINCR activity is triggered by SP1-zinc finger transcriptional factor which typically recognises the GC-rich sequences in promoter regions [68]. TINCR also increased proliferation and inhibited apoptosis in the BC cell lines, and this occurred because TINCR competed with miR-7. This interaction between TINCR and miR-7 also resulted in modulation of *KLF4* gene expression. The miR-7 ability of supressing BC metastasis through *KLF4* activity has previously been demonstrated [69]. Finally, experimental TINCR silencing suppressed proliferation in both in vivo and in vitro conditions [68].

Further TINCR mechanisms were comprehensively elucidated by Dong et al. [70]. These authors used SKBR-3-TR and BT474-TR trastuzumab-resistant cell lines, followed by mouse xenograft analysis. TINCR knockout decreased these cells’ chemoresistance, their EMT potential and EMT marker expression. The TINCR-lncRNA acted there as a ceRNA and inhibited activity of miR-125b which suppresses BC progression by regulating *HER2* expression [71]. miR-125b was also found to target Snail-1 which is an important EMT and chemoresistance regulator. Thus, TINCR regulated the miR-125b-HER2/Snail-1 pathway and this resulted in trastuzumab resistance and EMT induction. The noted TINCR up-regulation was considered due to abnormal H3K27 acetylation of the TINCR promoter region. The final part of Dong et al.’s study [70] concentrated on qPCR analysis of 60 HER2+ BC tumour tissues from 30 chemoresistant and 30 chemotherapy-sensitive patients, and results indicated higher TINCR activity in chemoresistant patients, who subsequently experienced worse overall survival rate.

That work was then complemented by Guo et al.’s recent research [72], which revealed TINCR effect in MCF-7 and MDA-MB-231 BC cell lines. TINCR over-expression there inhibited miR-589-3p activity because TINCR acted as the miR-589’s ceRNA. This resulted in higher cancer cell proliferation, migration and invasion and also inhibition of cancer cell apoptosis. In addition, experimental miR-589-3p over-expression partly inhibited the TINCR effect on tumourigenesis.

The TINCR BC action can strongly participate in increasing IGFR/AKT pathway activity, because both of these components are miR-589-3p target genes [73,74]. IGFR is a well-known trans-membrane tyrosine kinase receptor important in cell mitosis, proliferation, differentiation, apoptosis and regulation of Akt pathway activity [73,74]. Finally, Guo et al. [72] determined that higher TINCR expression was associated with poor survival rate in a cohort of 68 patients.

#### 3.1.3. MALAT lncRNA

lncRNA MALAT (MALAT) is abnormally expressed in several BC types and this abnormal expression correlates with poor prognosis and metastasis [75,76]. It is further indicated that this lncRNA activity can be inhibited by high 17-β oestradiol concentration [77]. One interesting study found that MALAT was elevated in patients with early post-BC-resection fever [78] and this led to worse prognosis. In addition MALAT knockout in mouse 4T1 xenografts significantly decreased inflammation and reduced the lung metastases so often seen in BC [78].

miR-1 was noted to directly target MALAT1 and small GTPase *CDC42*. The MALAT binding with miR-1 resulted in over-expression of Cdc42 which then promoted BC cell line migration and invasion [79]. MALAT1 therefore functioned as a ceRNA of *CDC42*. Moreover, MALAT action also inhibits E-cadherin expression and induces vimentin expression, and this leads to higher epithelial mesenchymal transition ratio (EMT). This directly contrasts with miR-1 action [79]. MALAT1 was also demonstrated to bind to miR-204, and there is reciprocal repression between miR-204 and MALAT1. In addition, E-box binding homeobox 2 (ZEB2) is a direct miR-204 target. ZEB2 is a very important factor in EMT promotion because it has been reported to inhibit E-cadherin and down-regulate a distinct set of constituents of desmosomes and adherens, gap and tight junctions [80,81].

#### 3.1.4. NEAT1 lncRNA

NEAT1 is an important oncogene in cancer and significantly affects EMT induction in BC [82]. Abnormal NEAT1 activity affected chemoresistance and cancer cell stemness in a cohort of 179 BC patients and it was expressed 6.86 times more in BC patients than in 192 controls [83]. NEAT1’s abnormally high expression was also instrumental in influencing tumour size, lymph node metastases and overall survival in a cohort of 40 BC patients [84].

There were also changes in EMT marker levels following NEAT1 knockout in various cancer cell lines. This particularly applied to E-cadherin up-regulation and β-catenin and N-cadherin down-regulation following NEAT1 inhibition [84]. Li et al. then indicated that NEAT1 activity in BC is largely oestrogen-inducible and NEAT1 mediated the interaction between FOXN3 and SIN3A in oestrogen-dependent BC [85]. The FOXN3-NEAT1-SIN3A complex is considered to suppress genes which inhibits EMT, especially *GATA3* [85], and connections between abrogated *GATA3* and increased EMT have previously been demonstrated [86]. In addition, *NEAT1* and *FOXN3* over-expression results in decreased epithelial markers including E-cadherin, α-catenin and γ-catenin, and it corresponds with diminished GATA3 expression. This also induces mesenchymal markers such as fibronectin and vimentin. This study highlighted the FOXN3-NEAT1-SIN3A effect on EMT promotion, MCF-7 cell line invasion and higher dissemination and metastasis under in vivo conditions in BC mouse models [85]. Finally, the authors also demonstrated higher NEAT1 and FOX3 expression and lower GATA3 expression in 24 BC patient tissue specimens [85].

A further important BC feedback loop includes NEAT1, miR-124 and STAT3. The NEAT1 and STAT3 expression levels were elevated in BC tissues, and this correlated with decreased miR-124 expression levels. Moreover, NEAT1 over-expression markedly increased STAT3 protein expression levels, but this effect was reversed by miR-124 over-expression. The inhibitory effects of miR-124 over-expression were also abolished by STAT3 over-expression. Finally, STAT3 silencing inhibited NEAT1 transcription in BC cells. Therefore, NEAT1 and STAT3 form a feedback loop via sponging miR-124 to promote BC progression. NEAT1 modulation which regulates STAT3 appears very promising as a potential therapeutic approach, because STAT3 is a major BC marker with many upstream regulators and downstream targets which are known to promote BC malignancy and strong chemo-resistance [87].

NEAT1 also acts as an ceRNA inhibiting function of miR-211 [88] which is down-regulated in cancer and considered a tumour-suppressor [89,90]. This miRNA also binds to *HMGA2* which regulates a network of transcription factors that drive EMT [88]. NEAT1 down-regulation ultimately decreases expression of the *HMGA2*, and this NEAT1/HMGA2 activity is in direct proportion. NEAT1 activity has also been experimentally inhibited by sh-NEAT1, and this resulted in decreased 5-FU resistance. NEAT1 activity can therefore affect chemo-resistance, and further analysis of this interaction could prove beneficial.

#### 3.1.5. DANCR lncRNA

DANCR lncRNA affects the activity of the EZH2 enzymatic subunit of the PRC2 polycomb repressive complex [91]. The PRC2 complex changes histone methylation status and regulates EMT-inducers transcription [92] and its abnormal activity is associated with various oncological diseases [93]. Research suggests that DANCR most likely has tumour-suppressive function because of its low activity in BC cell lines and tissues [91]. DANCR’s interaction with EZH2 is then thought to facilitate CD1 and EZH2 binding, and this causes EZH2 ubiquitination and degradation. DANCR therefore negatively regulates EZH2 action. While DANCR over-expression in mouse xenograft tumours formed from the injection of aggressive MDA-MB-231 cells decreased metastasis formation and spread, its knockout under in vitro conditions in MCF10A cells induced EMT, cell migration and invasion [91].

Zhang et al., however, record different DANCR and EHZ2 interaction [94]. Their study shows that DANCR lncRNA is more highly expressed and is considered to induce oncogenesis. DANCR did not initiate EZH2 degradation but promoted its binding to the *SOC3* gene promoter region, and this resulted in inhibited *SOC3* gene expression. This mechanism was observed under in vitro conditions in MCF-7, T47D, MDA-MB-231, and MDA-MB-468 cell lines. Finally, shDANCR knocked out DANCR in mouse xenografts with injected MDA-MB-231 and MDA-MB-468 cell lines, and this significantly reduced the number of metastatic BC nodules in mice lungs.

A further study demonstrated that DANCR was highly expressed in MCF-7 and MDA-MB-231 cell lines [95]. However, a different action mechanism was noted in this study. DANCR was demonstrated as a ceRNA targeting miR-216-5p and its knock-out increased E-cadherin and decreased *Nanog*, *OCT4*, and *SOX2*. The patients in this study were divided into DANCR expression groups, where patients with lower DANCR expression had slower disease development and better survival rates.

The DANCR-miR-758-3p-PAX6 molecular network is also affected in BC [96]. DANCR was again observed to be more highly expressed in both BC cell lines and tissues, and miR-758-39 was significantly down-regulated. However, experimental DANCR down-regulation in BC cell lines inhibited cell malignancy and induced apoptosis by increasing typical apoptotic markers. In addition to DANCR inhibiting miR-758-3p activity, it also regulates the activity of *PAX6*, which is a transcriptional factor abnormally expressed in BC, with subsequent poor prognosis [97].

#### 3.1.6. lncRNAs with Abnormal TNBC Expression

Recent studies have assessed the effect of lncRNA dysregulation in TNBC because this subtype is very aggressive and has poor prognosis [98]. Aughoff et al. [99] revealed that lncRNA LOC554202 is down-regulated in TNBC due to altered promoter region methylation, and this lncRNA includes a sequence encoding miR-31 which has a tumour-suppressive effect on BC formation and metastasis [100].

Additional research by Koduru et al. [101] employed freely available small RNA sequencing data from 24 TNBC patients and 14 healthy controls. They then used this in advanced data assembly and statistical analysis [101]. Their results suggested that up to 258 lncRNAs can be abnormally expressed in TNBC tissues under the less stringent conditions of *p* < 0.05. However, this decreased to 61 aberrantly expressed lncRNAs when *p* < 0.01 was applied. It was further determined that SC5DL, PURA, EIF2C2 and ELP4 were the most important of the up-regulated lncRNAs, and PAPLN, FLT3LG, NEK8, FLOT2 and ZNF75D were the most significant of the 33 remaining down-regulated lncRNAs. Data for stage-wise lncRNA profiling assertion were then analysed. This confirmed that 160 lncRNAs were in BC Stage I, 155 in stage II, and 79 in stage III. There were 79 lncRNAs concurrently regulated in stages I and II, three in stages I and III, and only one in stages II and III. This indicates how individual lncRNA abnormal activity can affect TNBC progression, but further study is required to elucidate these relationships.

The small nucleolar RNA host gene 12 (SNHG12) lncRNA is abnormally expressed in several BC cell lines [102] and this lncRNA’s pro-tumour effect was also found in TNBC tissues where higher expression correlated with tumour size and lymph node metastasis [103]. Research has established that SNHG12 is a direct transcriptional target of c-MYC, and that c-MYC activation of SNHG12 resulted in higher *MMP13* activity. It is therefore highly likely that *MMP13* is also a target of SNHG12. The higher SNHG12 rate in TNBC tissue was also directly proportional to *MMP13* activity rate.

*MMP13* is a metalloproteinase whose higher activity is typical in BC and its abnormal activity was initially identified in TNBC tumours [104]. *MMP13* degrades the extracellular matrix (ECM), but the process must be strictly regulated within physiological range. Moreover, an abnormal matrix is formed in tumour tissue when *MMP13* and other matrix metalloproteinase activity is too high, and this leads to greater invasion, metastasis and worse prognosis [104]. However, SNHG12 expression in BT-549 and MDA-MB-231 cell lines was down-regulated by siRNA induced c-Myc depletion, with subsequent diminution of adhesion ability and cell proliferation [103].

qPCR analysis determined that small nuclear NF90-associated (snaR) lncRNA has more than 15-fold expression in TNBC cell lines compared to controls [105]. Consequently, Snar lncRNA knockdown significantly decreased cancer progression in triple negative MDA-MB-231 cell lines. Further research demonstrated that snaR activity is significantly influenced by NRON lncRNA, and the expression of these two lncRNAs is in indirect proportion. In particular, snaR expression increased from BC stage I to IV, while NRON expression decreased with increasing clinical staging [106].

#### 3.1.7. Additional lncRNAs

(1). The BCRT1 lncRNA is abnormally expressed in BC, where it functions as a ceRNA and competitively binds with miR-1303 to prevent degradation of its *PTBP3* target gene which is an important BC promoter [107]. It is further considered that lncRNA BCRT1 can promote M2 polarisation, and that this lncRNA expression can be induced by direct hypoxia inducible factor-1a (HIF-1α) binding to hormone-responsive elements in its promoter region [107].

(2). Chen et al. [108] recorded the association of HIF-1α up-regulation with lncRNA action and BC formation. These researchers found that tumour associated macrophages (TAM) produce extracellular vesicles (EV) which contain HIF-1α-stabilising long-noncoding-RNA (HISLA). This HISLA lncRNA was naturally transmitted from EV to tumour cells where it blocked HIF-1α interaction with prolyl hydroxylase domain 2 (PHD2). This interaction prevented physiological HIF-1α degradation, and the subsequent rise in protein level increased cellular aerobic glycolysis [109]. Experiments have also revealed that lactate released from glycolytic tumour cells up-regulates macrophage HISLA, and blocked transmission between EV and TAM results in glycolytic inhibition and decreased chemoresistance. Interaction between macrophages and tumour cells can therefore significantly influence tumour development, and modification of this interaction appears a worthy treatment target.

(3). The LINC0178 lncRNA can have an oncogenic effect in BC, because it influences tumour-suppressor miR-125 [110] by inhibiting its binding to the Dicer complex and thus repressing miR-125 maturation.

(4). RP1-506.5 lncRNA is abnormally expressed in BC [111]. This lncRNA represses the important p27Kip inhibitor of cyclin dependent kinase 1B due to formation of the RP1-p-4E-BP1/eIF4E complex. This complex precludes interaction of eIF4E and eIF4G eukaryotic translation initiation factors which normally activate p27Kip translation. The RP1-506.5 lncRNA also reduces *Snail1* gene activity, and it is further demonstrated that the *KLF5* gene affects this lncRNA’s activation. Finally, abnormal activity of the *KLF5* transcription factor and subsequent activation of its target genes have previously been associated with rapid BC growth and poor prognosis [112].

(5). HOTAIR lncRNA is transcribed from the intergenic region of the *HOXC* gene and this lncRNA activity dysregulation occurs in both ER positive and negative cancers with metastasis formation and worse disease course [113,114,115]. HOTAIR is regulated by abundant oestrogen receptors and response elements in ER-positive BCs, and its activity is in direct proportion to oestradiol levels [116]. Although the mechanisms of HOTAIR lncRNA’s increased activity in ER negative BCs is less understood, oestradiol involvement has been established also in this case [117]. Oestradiol binds to the G-protein-coupled oestrogen receptors (GEDPR) in these tumours, and this triggers a cascade which culminates in inhibited miR-148 transcription [118]. It is important that miR-148 is also a direct HOTAIR inhibitor. Therefore, the increased HOTAIR expression in ER- BC is not caused by direct initiation of transcription, as occurs in ER positive BC, but due to a loss of function of its inhibitor miR-148 [117].

#### 3.1.8. lncRNAs as Biomarkers in Liquid Biopsy

The important H19, NEAT1 and HOTAIR lncRNAs associated with BC development were analysed for detection of early-stage BC in patient plasma samples [119]. While lncRNA H19 expression in plasma samples correlated with expression of its target miR-675 in both healthy and cancer-afflicted patients, the expression of NEAT1 and its target miR-204 correlated only in HER2+ patient plasma samples. In contrast, although HOTAIR could not be detected in plasma samples, its target miR-331 was detectable, and this was associated with nodal status. This miRNA was also differentially expressed in BC patient samples compared to samples from healthy subjects. Finally, all three lncRNAs were aberrantly expressed in MCF-7 cell lines, and their expression levels were in indirect proportion to their target miRNA levels. Table 1 lists the abnormally expressed lncRNAs and their BC targets.

## 4. Piwi-Interacting RNAs

The piRNAs are 26–31 nucleotides long and associated with PIWI proteins which are a clade in the Argonaute protein family [120,121]. Piwi indicates “P-element–induced wimpy testes’, and their presence in eukaryotic cells was discovered in mouse sperm cells in 2006 [122]. While their initial presence in human cells was demonstrated in germline and stem-cells [123], they are also present in differentiated somatic cells, but in significantly smaller numbers [120,121]. However, over 30,000 piRNAs are currently described in the eukaryotic genome [122].

**Figure 3:**
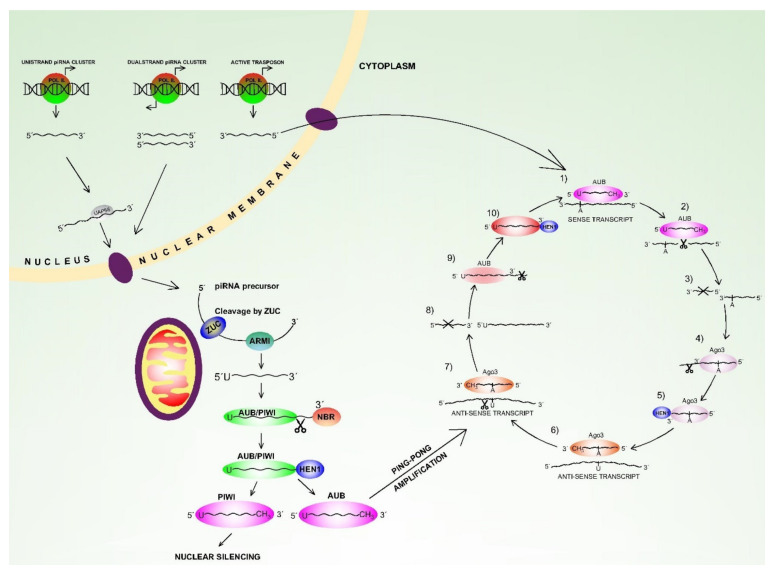
Primary and secondary biogenesis of piRNAs in model *Drosophila melanogaster* germline cells. The piRNA precursors can be transcribed from uni-strand and dual-strand piRNA clusters by polymerase II. Majority of these precursors are antisense (5′-3′) relative to transposon transcripts. Export from nucleus to processing sites is mediated by UAP-56 activity. The piRNA precursors are resolved by Armitage (armi) RNA helicase after export, and this leads to their unwinding. The 5′end processing is then mediated by the Zucchini mitochondria-associated nuclease (ZUC). ZUC action transforms the piRNA precursors into pre-piRNAs which are subsequently loaded into Piwi or AUB protein complexes. Here, fragments with Uracil bias at the 5′end are primarily selected. The overhanging 3′end is trimmed with 3′ to 5′ Nibbler exonuclease (Nib), Hen1 then methylates the 3′end and the piRNAs are then mature. piRNAs loaded into the Piwi protein are then involved in transcriptional gene silencing (TGS) in the nucleus. In contrast, the Aub–piRNA complex triggers the ping-pong amplification pathway by recognising and cleaving transposon mRNA. The product of this cleavage is converted into new sense oriented piRNA (secondary piRNA) which has a 10A bias, and this is subsequently loaded into the Ago3 protein complex and trimmed and methylated. The Ago3-piRNa complex similarly recognises and cleaves the anti-sense cluster transcript, and the product of this cleavage re-initiates the cycle. This provides one-cycle transposon sequence cleavage and simultaneous amplification of the piRNA sequence. The ping-pong amplification is therefore a mechanism of post-transcriptional gene silencing (PTSG).

The main piRNA role is silencing of transposable elements (TE) in the germline cells at both transcriptional and post-transcriptional levels. Although this function is conserved in most animal species, different proteins and mechanisms can be involved [120,121,122]. Post transcriptional gene silencing (PTGS) of TE elements occurs in the cytoplasm, where the target transcript is sliced, and cleavage-competent Piwi-proteins are required for this processing. The best described PTGS mechanism is the “ping-pong-cycle” described later herein (Figure 3). Transcriptional silencing (TGS) occurs in the nucleus where the piwi-piRNA complex recognises the nascent TE transcript by complementarity and this is followed by silencing thought the interaction with the heterochromatin silencing machinery. This results in H3K4me2 removal and H3K9me2/3 deposition, and this is followed by HP1a and histone H1 covering the target locus to maintain the repressed status [124]. However, many of the molecular mechanisms of this process remain unknown and require investigation. Moreover, piwi-guided transcriptional silencing induces promoter DNA methylation changes in the mammalian germline [125]. The piRNA-PIWI complex can activate or repress translation in early mouse spermatogenesis and affect mRNA degradation in a microRNA-like manner at later stages [126]. The complex of piRNAs, Hsp90 and Hop protein are also involved in “canalisation” which ensures robust development [127] and although piRNAs activity is mainly associated with germline cells, it also has the following functions in somatic cells; (1) piRNAs influence *CREB2* promoter methylation in nematodes and this affects long term memory formation [128]; (2) involvement in dendritic spine development in the mouse hippocampus [129]; (3) mediation of Nanos mRNA de-adenylation and degradation. The correct Nanos level is essential to form the Drosophila embryo anterior-posterior axis [130]. In addition, it is presumed that piRNA function in somatic cells is ancestral and lost in some species [120,121].

piRNAs are transcribed from ‘piRNA clusters’ which are usually over 100 kb long and consist mostly of transposable elements, but also contain different repetitive regions accumulated during evolution [120,121]. A smaller number of piRNAs are transcribed from inter-genic non-coding regions and protein-coding 3′-UTR gene regions [122,123], and some studies suggest they can be transcribed from tRNA and snoRNA sequences [122,123]. The piRNA clusters can be uni-strand and dual-strand. Uni-strand clusters produce piRNA precursors from only one genomic DNA strand. They harbour H3K4me2 marks, and the piRNA precursors transcribed from uni-strand clusters are both 5′-capped and 3′-polyadenylated. In contrast, the dual-strand clusters produce piRNA precursors mapping to both genomic strands. They do not have similar marking, and their transcribed precursors are only 5′capped [122,123]. The piRNA precursors are transferred from the nucleus after transcription and undergo primary biogenesis. Although the precursors are predominantly anti-sense (5′-3′) to the target TE sequence, this is not an absolute rule. For example, the mouse piRNA precursors have sense polarity (3′-5′).

These precursors are then shortened and bind to specific PIWIL1/HIWI, PIWIL2/HILI, PIWIL3 and PIWIL4/HIWI2 Piwi proteins or Aubergine (Aub). Here, the HIWI indicates human protein, in order to distinguish it from non-human protein. Multi-step processing then occurs to give the final PIWI/AUB-piRNA complex, followed by methylation of the piRNA 3′ end by HEN1 methyltransferase. Finally, the entire complex enters the nucleus for transcriptional silencing or, alternatively, it is involved in secondary biogenesis (ping-pong cycle) [122,123,131,132] (Figure 3).

A summary of the entire process is as follows; an initial pool of piRNAs is created in primary biogenesis and this targets multiple TEs. This is followed by amplification of RNA sequences that target active transposons in secondary biogenesis and these then repress transposons via slicer-dependent PTGS (Figure 3).

The Aub-piwi complex from primary biogenesis recognises and cleaves the cognate transcript of transposon mRNAs with opposite orientation, and the cleaved product is then converted into a new sense piRNA associated with Ago3.

Ago3 with incorporated piRNA is further modified and subsequently recognises and cleaves the cluster transcripts. The products of this slicing then re-initiate the cycle. This process ensures amplification of particular piwiRNAs and the degradation of both sense and anti-sense transposon transcripts [122,123]. Although the finer details of piRNA biogenesis have not been elucidated and most of our understanding comes from *Drosophila* germline cells, it is anticipated that the mechanisms in human cells are the same, or very similar, to those documented in drosophila [122,123].

Martinez et al.’s comprehensive study [123] highlights that although only approximately 1% of piRNAs is expressed in healthy somatic cells compared to germline cells, almost two-fold piRNAs over-expression occurs in cancer [123]. Their expression level there differentiates between healthy and tumor tissue [123], and level changes are associated with cancer cell migration and invasion [133,134]. It is therefore presumed that the normal physiological mechanisms of piRNA-induced target regulation in germlines become abnormal in cancer cells and contribute to their formation and development. This includes physiological abnormality in DNA methylation, histone modificiation, translation initiation and inhibition and mRNA degradation. The connection between abnormal piRNA activity and the development of various cancers has previously been documented [122,135,136].

### 4.1. Piwi-Interacting RNAs and BC

The presence of piRNAs and their altered function in tumour cells was first demonstrated in 2010 in the well-known HeLa cells [137]. The piRNA effect on tumorigenesis by binding to the target mRNA or altering epigenetic status was then demonstrated in several tumours, especially in lung cancer [138,139] and colon cancer [140].

The initial association between abnormal piRNAs activity and BC tumorigenesis was demonstrated in 2011 in incidental research by Cheng et al. [141]. Although their work focused on the abnormal activity of piRNA-651 in gastric cancer tissue, the authors then recognised that this piRNA is also up-regulated in FFPE samples from BC. The piRNA-651 up-regulation in tumorigenesis has since been repeatedly demonstrated in vitro, and it especially contributes to increased proliferation and migration and inhibited apoptosis [142,143,144]. The piRNA-651 is also considered to up-regulate cyclin D1 and CDK, but the precise mechanisms require elucidation [143].

Huang et al. [145] produced the first study focused exclusively on multiple piRNAs presence in BC tissues. These researchers performed deep sequencing of 4 tumour samples and five controls, and results were subsequently verified by qPCR analysis of 50 samples. Deep sequencing revealed piRNA-4897, piRNA-19825, piRNA-20365, piRNA-20485, and piRNA-20582 were up-regulated, and that piRNA-17485 was down-regulated. Similar results were demonstrated by qPCR except for piRNA-19825. Finally, the piRNA-4897 abnormal expression there was associated with lymph-node positivity.

The piRNA expression in MCF-7, SKBR3 and ZR-75.1 BC cell lines is affected by cell cycle phase and oestrogen receptor activity [146]. Research results documented that 39 piRNAs were differentially expressed in exponentially growing cells compared to senescence cells, and 25 piRNAs had different expression-levels in ERβ+ and ERβ- cells [146]. The authors subsequently performed RNA sequencing analyses on four paired BC tissues. The abnormal expression of almost 150 piRNAs was revealed under less strict *p* < 0.05 statistical conditions, and subsequent *p* < 0.01 statistical analysis determined that the eight PiRNA-34736, PiRNA-36249, PiRNA-35407, PiRNA-34377, PiRNA-36318, PiRNA-36026, PiRNA-31106 and PiRNA-36743 piRNAs have statistically significantly higher activity. This study also determined that active piRNAs in somatic cells made up only 1% of those active in germline cells [146].

#### 4.1.1. piRNA Effects on Expression of Other Genes

There was significantly higher piRNA-932 activity in cells with high metastatic potential, and especially in those with induced EMT [147]. The authors consider that piRNA-932 forms immune complexes through precipitation with piwil2, and that the complexes will subsequently affect latexin gene expression by hyper-methylation of its promoter region. Latexin is a tumour-suppressor and negative stem cell regulator which induces removal of old stem cells and prevents their eventual transformation into tumour cells [147].

Further piRNA effects indicate that piRNA-021285 significantly influences *ARHGAP11A* gene promoter methylation level [148]. This gene encodes the Rho GTPase-activating protein which is considered oncogenic [148], and the study demonstrated that MCF-7 cell lines mimic-transfected with piRNA-021285 mutational variant have significantly lower *ARHGAP11A* gene promoter region methylation levels compared to piRNA-021285 wild type mimic-transfected MCF7 cell. This was subsequently connected with higher expression of this gene and increased cell invasion. Importantly, the SNV rs1326306 G>T in piR-021285 was identified in a Connecticut-US sub-population and was strongly associated with increased likelihood of BC [148]. Finally, the *ARHGAP11A* gene was abnormally expressed in basal-like BC and it stimulated BC development in in vitro studies [149].

piRNA-36712 also has important functions in BC development [150]. In contrast to piRNAs with higher BC tissue expression, this piRNA’s activity was down-regulated in resected cancerous tissues and in MCF-7 and ZR75-1 cancer cell lines. The piRNA-36712 tumour-suppressive effect is therefore expected, and its expression rate in BC patients correlates with outcome. Those with lower expression levels have lower progression-free survival rates. This piRNA’s effect is related to its interaction with *SEPWP1* gene mRNA whose levels were increased following piRNA-36712 knockout. *SEPW1* is an important cell cycle regulator and its depletion increases p53 and p21 activity by suppressing their degradation by ubiquitination and this leads to G1 cell-cycle arrest [151,152]. Moreover, authors found that up-regulation of p53 reduced Slug and increased E-cadherin expression. This is supported by research that p53 can suppress cancer invasion by inducing MDM2-mediated Slug degradation. Slug is a transcription repressor that regulates E-cadherin expression [153,154].

In addition piR-sno 75, which is a snoRNA-derived piRNA has quite complex function in balancing BC epigenetic status [155]. piR-sno 75 has significantly lower expression in BC and experimental over-expression leads to TRAIL up-regulation. Here, piR-sno75 was shown to guide the recruitment of MLL3/COMPASS-like complex to the TRAIL promoter and this induced H3K4 methylation and H3K27 de-methylation and led to increased *TRAIL* expression and subsequent apoptosis induction. Table 2 summarises the abnormally expressed piRNAs and piwi proteins in BC.

#### 4.1.2. Abnormalities in PIWI Proteins in BC

The increased expression of Piwi proteins required for general piRNA function has also been reported in BC tissues. Piwil2 and Piwil4 are highly expressed in SKBR3 cell lines [146], and altered Piwil2 expression was demonstrated in 30.8% CD44+/CD24- cells cultured from resected tumour tissues. These cells form a fraction of the stem cells typically present in BC tumorous masses [156]. The Piwil2 expression was also significantly higher in BC than in para-cancerous tissues and hyperplasias, and its protein expression rate was associated with age, histological type, tumour stage and size, and lymph node metastasis. Moreover, metastasis occurred in over 50% of patients with abnormally expressed Piwil2. This was evident even after operative removal of the primary tumour, and this strongly contrasts with the metastasis noted in only 13% of patients with lower expressed Piwil2 [147].

## 5. Small Nucleolar RNAs

snoRNAs are short non-coding RNAs from 60 to 300 bp long, and most are encoded in intronic or other non-coding regions of genes encoding proteins involved in ribosome synthesis. The final snoRNA transcription is also intimately associated with host-gene expression [157]. The snoRNA processing commences in the nucleus. It continues in the cytoplasm, and it is necessary to incorporate them into the snorP protein complex. This process improves their stability so that they can be incorporated in the nucleus, usually in cajal bodies. Cajal bodies provide the major sites for splicing and final modification [158].

The two major snoRNA groups are C/D box and H/ACA box [157,159]. Their major function is ribosomal RNA modification and processing, with the following functional division; the C/D BOX snoRNAs act primarily as sequence-specific guides which direct rRNA modification by 2′-O-ribose methylation and the H/ACA group are especially involved in pseudo-uridylation of selected regions essential for ribosomal function [157,159]. However, further research established a broad spectrum of snoRNA functions; snoRNAs influence the modification of various cellular RNAs such as snRNAs and miRNAs [157], by affecting pre-RNA cleavage [9,160]. They are also considered to directly affect mRNA splicing and influence alternative splicing in serotonin receptor subtype 2C [161], and *DPM2*, *TAF1*, *RALGPS1*, *PBRM1*, and *CRHR1* pre-mRNAs [162].

Moreover, the expression of more than 200 genes in HEK 293T cells is altered in vitro by SNORD15 and SNORD16 RNA over-expression, and researchers presume direct association of these events [163]. The snoRNAs are also expected to regulate E2F7 transcriptional factor splicing [163], and abnormal snoRNA activity has been observed under oxidative stress from palmitate and hydrogen peroxide treatment [164]. The snoRNA U60 is involved in plasma-membrane cholesterol trafficking and reduces cholesterol synthesis [165]. Jinn et al. reported that snoRNA U17 influence on the hypoxia up-regulated mitochondrial movement regulator mRNA (HUMMR) also leads to modification of cholesterol synthesis and trafficking [166]. Finally, there is a reportedly important snoRNA involvement in the phosphoinositide 3-kinase (PI3K)/ACT, p53, and Wnt/β-catenin cell-signalling pathways [157].

In summary, although snoRNAs have diverse cell functions and mechanisms, these are not adequately elucidated. For example, there are also ‘orphan snoRNAs’ without rRNA modification function, which possess no sequence complementary to any known rRNAs and have no involvement in ribosomal biogenesis [167]. Elucidation of the precise functions of these orphan snoRNAs will add to existing knowledge of snoRNA mechanisms in healthy and tumorous cells.

### 5.1. SnoRNAs and BC

Increased ribosome biogenesis and nucleolar activity are associated with cancer development [168,169] and enlarged nucleoli are markers of tumour aggression [170]. However, the impact of snoRNA abnormalities in tumourigenesis was largely overlooked until recent research focused on their effects in this development.

#### 5.1.1. SnoRNAs Affect p53 Response

Su et al. [170] revealed the strong association between abnormally high expression of several snoRNAs and BC development in animal models and human tumours. The U15a, U15b, U22, MBI-43 and U87 SnoRNAs were abnormally expressed in animal models and HBII, U22, U3, U8, U15b, U94 and U97 in human tissues. These authors also reported the in vitro association of abnormal snoRNAs, p53, and fibrillarin (FBL) activity. Fibrillarin is an important enzymatic component which affects snoRNAs accumulation and higher snoRNA and FBL expressions correlate in direct proportion [171]. Fibrillarin mRNA and protein levels were up-regulated in 60% of human BC tumour tissues and its inhibition decreased the frequency of tumour formation and volume in mouse models. There was also reciprocal FBL interaction in MCF-7 cell lines [172], where FBL deletion increased p53 activity and its over-expression reduced p53 response. The experimental suppression of snoRNAs biogenesis also induced p53 dependent cycle arrest, and the authors therefore considered that p53 responds to snoRNA pathway inhibition. A summary of these multiple interaction indicates ‘vast cross-talk’ between p53, FBL and snoRNAs and modulation of this entire interaction system can be beneficial in improving BC treatment

Langhendries et al. demonstrated abnormally expressed C/D box snoRNAs U3 and U8 in BC cells [173]. Depletion of both these snoRNAs in MCF-7 cells resulted in a strong anti-tumour p53 stress response which led to higher p53 stability, cell cycle arrest and apoptosis. This was observed in MCF-7 BC cell lines and H1944 lung cancer cell lines. The tumourigenic potential of H1944 cells in mouse xenografts was significantly reduced after U3 suppression, and completely by U8 suppression. However, this experiment was not performed with BC cell lines, and outcomes are therefore doubtful, so follow-up analysis on BC cell lines would be efficacious.

#### 5.1.2. Association between Abnormal snoRNA and miRNA Expression

The RNU44, RNU48, RNU43 and RNU6B snoRNAs were variably expressed in various BC cancer types in a cohort of 219 patient [174]. This was associated with expression of miR-21, miR-210, and miR-10b, and this is important because miR-21 is a significant promoter of BC progression, proliferation and metastasis [175]. Altered snoRNAs expression was also associated with greater disease aggression. Moreover, RNU44 snoRNA is transcribed from the *GAS5* gene intronic region. This gene regulates cell cycle arrest in stress conditions and low RNU44 levels therefore mark poor prognosis. In addition, the RNU48 and RNU43 snoRNAs are transcribed from the RPL3 and C6orf48 cancer-associated genes [175].

#### 5.1.3. The Effect in BC of Micro-RNA-Like Fragments Arising from snoRNAs

Some snoRNAs can proceed to short and stable micro-RNA-like fragments. These are synonymously named sno-miRNA and sdRNA (‘small nucleolar RNAs-derived microRNAs’). sdRNA-93 is markedly over-expressed in MCF-7 and MDA-MB-231 BC cell lines and while sdRNA93 induces cell invasion, its inhibition prevents it [176]. Although the authors reported increased sdRNA-93 expression in Luminal B Her2+ tumour tissue, its expression was only minimally increased in healthy tissues and BC subtypes other than luminal B Her2+. In addition, sdRNA93 is involved in regulation of the *Pipox* gene whose protein product regulates sarcosine metabolism and alterations in this protein product are associated with BC metastasis formation [177].

Abnormal sno-miR-28 expression has been detected in BC [172] and this targets *TAF9B* which stabilises p53 in physiological conditions. A brief explanation of the processes involved here is that the, the interaction between p53, NHG1, sno-miR-28, and TAF9B results in a signalling cascade, which significantly affects p53 and modifies its down-stream gene network. Table 3 below lists the snoRNA actions.

## 6. Small Nuclear RNAs

snRNA are small non-coding RNAs in the Cajal bodies and splicing speckles in the nucleus [178,179]. The snRNAs are approximately 150 nt, and have the primary function of pre-mRNA post-transcriptional modification [178]. The following two main classes differ in common sequences and interacting proteins.

(1) the Sm-class comprise U1, U2, U4, U4atac, U5, U7, U11, and U12 snRNAs transcribed by RNA polymerase II and their processing involves transfer to the cytoplasm and return to the nucleus.

(2) the Lsm-class contains only U6 and U6atac. These are transcribed by polymerase III and remains in the cell nucleus [180,181].

The snRNAs unite with an approximately 150 proteins to form a major or minor spliceosome complex dependent on the snRNAs involved [182,183]; a major spliceosome complex which removes 99.5% of introns [178] is formed with U1, U2, U4, U5, and U6 snRNAs, and a minor complex with U4atac, U5, U6atac, U11, and U12. It is clear here that only snRNA U5 is present in both complexes [181]. Spliceosomal abnormality is evident in the following pathologies; the congenital abnormalities that occur in dwarfism [181] and neurodegenerative diseases such as Alzheimer [184] Somatic spliceosome abnormalities are typical in the snRNA component mutations responsible for pre-cancerous and cancerous lesions [180,181]. An example of this is highlighted in in vitro experiments which reveal that snRNA U1 over-expression causes altered expression levels in over 900 genes, and 73 of these are directly involved in 12 important cell signalling pathways directly associated with various cancer development [184].

### SnRNAs and BC

Although aberrant mRNA splicing occurs in many cancers [185], oncology research usually addresses only altered function and activity of the “regulatory” factors which control splicing, and there has been minimal analysis of snRNAs as “basal factors” required for catalysing the splicing process. However, recent research by Dvinge et al. [186] content that varied snRNA abundance in different BC types is not random and most BC samples have subtype-specific snRNA expression patterns.

These researchers also considered that TNBC could be divided into two subtypes based solely on snRNA levels, and that splicing changes predominantly affected only one exon after snRNA knock-down in MCF-7 cells. They subsequently performed deep sequencing of 136 invasive ductal carcinomas to compare naturally occurring splicing anomalies with those associated with snRNA knock-down in MCF-7 cells. The results confirmed that splicing anomalies accorded with snRNA expression, and that the observed abnormal splicing was mostly snRNA related. There was also confirmed relative and absolute snRNA expression variability in a wide range of biological conditions and in healthyand tumorous tissues.

The U1 snRNA exerts significant impact on migration and invasion in BC cell lines [187]. While shortening of the mRNA 3-UTR region occurs from altered proximal polyadenylation signals (PAS) in introns and the last exon [188,189,190], PAS activity can be silenced by snRNP U1 [191]. These shorter mRNA isoforms are typical in immune cells, neurons, and some cultivated cell lines, and they are also present in various cancers including BC [188,189,190]. Research has highlighted that U1 inhibition results in premature transcription termination and mRNA shortening in BC [187], but U1 over-expression not only negates these effects but significantly decreases cell line ability to migrate and spread. This U1 snRNP therefore presents a suitable target for inhibiting BC spread.

Although snRNAs are typically situated in the nucleus, they can be detected by liquid biopsy and potentially used for early non-invasive cancer detection [192]. For example, higher snRNA U6 levels have been found in BC patient plasma samples [193]. This is typical in both active and inactive disease and in ER+ and ER- BC subtypes, but not in healthy women. The permanently elevated U6 levels also indicate increased polymerase III activity in BC, regardless of disease progression [193]. Table 4 lists snRNA actions in BC.

## 7. Small-Interfering and Short Hairpin RNAs

The double-stranded small interfering RNAs (siRNAs) with 20–25 nt were first discovered in *Caenorhabditis elegans* [194,195] and then in plants and invertebrates [196]. The siRNAs’ function in plant, fungal, unicellular and invertebrate cells is regulation of post transcriptional expression and protection against viral elements [197]. The siRNAs in these taxa can also originate as exogenous transcripts derived from viral elements [194]. It remains debatable whether siRNAs can be naturally present in higher eukaryotes, but some studies also admit this possibility [198]. However, the presence of dsRNAs in higher eukaryotes cells leads to interferon response [199].

Artificially prepared siRNAs are often used studies in the expectation of modulating individual gene activity. This would be therapeutic in cancer and in diseases where conventional treatment is insufficiently effective [194,200]. However siRNAs cannot passively diffuse through cellular membranes [201,202,203] and their application is therefore limited by delivery to the cell target area. This remains a challenge because siRNAs are rapidly degraded by ubiquitous endo- and exonucleases, and administrative, vascular, cellular and immune barriers must be overcome for effective siRNA delivery. Additional requirements are that siRNAs must be chemically modified for increased stability and their length must be limited to 30 nt [204].

Finally, the siRNA delivery system can be viral or non-viral [201], where the non-viral delivery systems are predominantly lipid-based, polymer-based or utilise nucleotide-derived nanoparticles [194,201,202,203,204].

**Figure 4:**
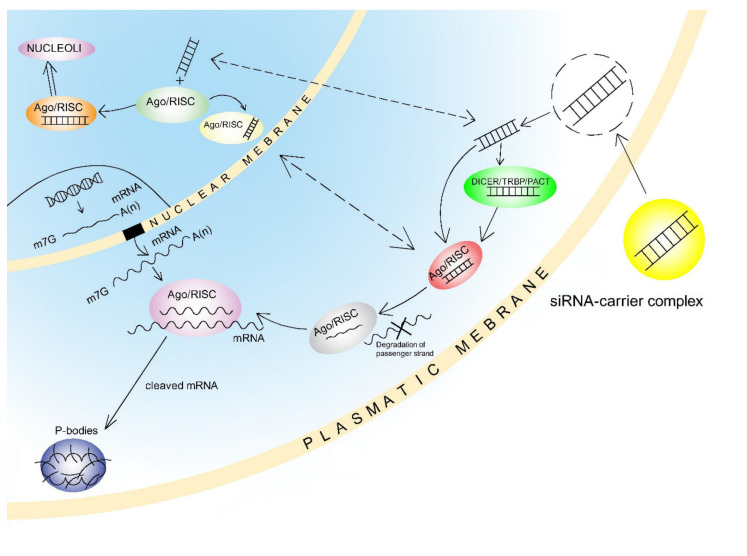
siRNA action mechanism. Following delivery to the cytoplasm, the siRNA’s are directly loaded into the RISC complex or they undergo Dicer-mediated processing before loading in the RISC complex. Guide strand selection and passenger strand degradation depend on the several properties The guide strand has weaker binding at the 5′-end, is U-biased at that end and also has excess purines. The Ago/RISC complex then recognises the target mRNA and this is cleaved and degraded, or its translation is suppressed by sequestration in P-bodies. The presence of both individual siRNAs and those loaded in the Ago/RISC complex in the transfected cells’ nucleus has been noted, and there is also shuttling of this complex between cytoplasm and nucleus. The precise mechanisms of these actions require elucidation. Edited with permission from [209].

Following delivery of artificially prepared siRNA to the cytoplasm, the siRNAs are either loaded directly into the RISC complex or processed by the Dicer complex before loading. The strands are subsequently separated, and the RISC complex is directed to a specific mRNA depending on the guide-strand. Ago-2 then cleaves the target mRNA between bases 10 and 11 relative to the 5′ end of the siRNA antisense strand and mRNA is degraded. There can also be alternative action by different Ago proteins which do not have catalytic activity, and this leads to translation repression through mRNA sequestration in processing bodies (P-bodies) [194,205] (Figure 4).

In addition, intact siRNAs have been reported present in the nucleus of transfected cells, and also to remain there for at least 15 min [206]. Moreover, while the Ago/RISC complex loaded with siRNA shuffled between nucleus and cytoplasm [207], the mechanism for this process has not been elucidated. Recent research indicates that Ago2 loaded with small RNA and localised in the nucleus can regulate both miRNA and lncRNA transcription levels [208].

**Figure 5:**
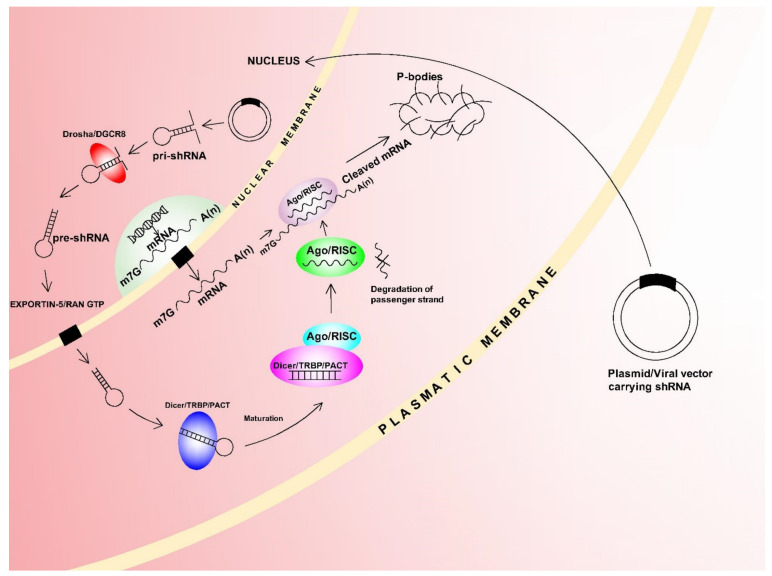
**shRNA mechanism action**. shRNAs must be encoded in an appropriate expression vector for delivery to the nucleus for transcription. The shRNAs are transcribed by either RNA polymerase II or III, depending on the promoter driving their expression. The pri-shRNA primary transcript is recognised by the Drosha/DGCR8 complex and processed to precursor pre-shRNA. These shRNAs are then transported into the cytoplasm via Exportin 5, loaded into the Dicer/PRBT/PACT complex and processed to mature shRNAs. The shRNAs in the DICER complex then associates with the Ago/RISC complex, and this results in mRNA cleavage and degradation or suppression of mRNA translation. Edited with permission from [209].

The 20–25 nt short hairpin RNAs (shRNA) are artificially prepared, and these can also be used to alter gene expression. However, in contrast to siRNAs which must be delivered to the cytoplasm, shRNAs must be delivered by appropriate plasmid or viral vector to the nucleus for transcription, and the primary transcript (pri-shRNA) is then processed by the Drosha complex. Precursor shRNAs (pre-shRNAs) are formed as a result of this processing, and these are incorporated in the Dicer complex after Exportin-5-mediated transport from the nucleus. The shRNA hairpin loop is excised by the Dicer complex and this then forms an active double-stranded siRNA with 2nt 3′ overhangs which is subsequently incorporated in the Ago/RISC complex. The action mechanisms which then lead to mRNA cleavage or translation repression are then the same as in siRNAs [209] (Figure 5).

### 7.1. SiRNAs and BC

#### 7.1.1. siRNA Based Inhibition of BC Associated Pathways

Researchers have endeavoured to decrease BC growth using siRNAs which inhibit abnormally active and typically dysregulated signalling pathways such as NF-κB [210]. The application of micelleplex containing siRNA-p65 and cisplatin-prodrug inhibited this pathway in 4T1 BC murine cells [211]. P65 is an essential protein, involved in NF-κB heterodimer formation, nuclear translocation and activation [212]. The application of micelleplexes containing siRNA-p65 was first successfully applied in cell lines and has subsequently proven efficacious in in vivo T4 mice xenograft tumours. This application inhibited tumour growth-rate compared to controls. In addition, the siRNA-p65 mediated silencing of p65 protein decreased in 4T1 cell lines *MMP9* expression by 40% and cyclin D1 expression by 70%.

Frequent abnormalities in the Wnt/β-catenine/EMT pathway were also noted in BC tissues [213]. The DANCR lncRNA is known to have significant impact on this pathway, and Vaidya et al. therefore examined siRNA DANCR inhibition [214]. Their application of RGD-PEG-ECO/siDANCR nanoparticles produced up to 90% DANCR knockdown in MDA-MB-231 and BT549 cancer lines within seven days. At the molecular level, decreased PRC2-mediated H3K27-trimethylation and altered phosphorylation profiles of several kinases occurred after DANCR inhibition. In addition, application of the above nanoparticles in TNBC mouse xenografts suppressed tumours without side effects.

Abnormalities in the VEGF pathway cause rapid angiogenesis, and this can hasten cancer development, including BC [215]. VEGF pathway abnormality in BC is also connected with increased lymphangiogenesis, which can result in aggressive spread to the lymph nodes and even metastases in the lungs [216]. Feng et al. demonstrated that vapreotide-modified core-shell type nanoparticles which co-encapsulated VEGF targeted siRNA and paclitaxel can be effectively transported into BC cells via samostatin receptors. This resulted in siRNA interference which reduced VEGF activity in MCF-7 cell lines [217], and the nanoparticle delivery to mouse xenografts resulted in significantly decreased tumour vascularity and growth.

#### 7.1.2. siRNA-Based Inhibition of Cell Cycle Regulators and Transcriptional Factors in BC

Polo-like kinase 1 (PLK1) is a serine-threonine kinase, a trigger of G2/M transition involved in centrosome maturation [218]. *PLK1* is considered a proto-oncogene which induces centrosome abnormalities and cell-cycle progression defects [218]. Further, PLK1 phosphorylates BRCA1 thus impairing its involvement in homologous recombination [219], and reported *PLK1* expression is higher in TNBC than in both healthy cells and benign tumours [220]. Moreover, PLK1 inhibition by BI-2536 siRNA leads to G2/M cell-cycle arrest and apoptosis initiation in the MDA-MB-231, Hs578T, MDA-MB-436, MDA-MB- 468 and HCC1937 BC cell lines [220].

The PLK-inhibiting siRNA-siPLK1 delivered to TNBC cell lines in meso-porous-silica nanoparticles reduced all cell line viability [221] and decreased *PLK1* mRNA levels by 69 to 87% and protein levels by 64–91%, depending on cell lines. In addition, meso-porous-nanoparticles lacking incorporated siRNA should have ROS scavenging ability, and empty particle application consequently resulted in reduced NOX4 expression and an overall decrease in cellular ROS. Finally, BC metastasis to lungs has been reported in animal models injected with LM2-4lucþ/H2N cells, and the application of the meso-porous-silica nanoparticles with siPLK1 resulted in 80% mRNA knockout and approximately 90% decrease in tumour size [221].

Nachreiner et al. [222]. investigated siRNA-inhibition of *PLK1* and the *EEF2*, *GRK4* and *SKIP5* genes. Here, siRNAs were applied to MCF-7 cells using the highly specific HER3 aptamers A30. These characteristically bind to the human epidermal growth factor receptor-3 extracellular domain, and this domain is routinely over-expressed on the BC cell surfaces [222]. This siRNA application reduced cell viability and also the expression of all analysed genes at the mRNA level. In contrast, there was no loss of cell viability in controls.

Further research showed that RNase-resistant nanoparticles suppress *XBP1* gene activity in mouse HER2+ BC [223]. Silencing this gene by 3WJ-HER2apt-siXBP1 nanoparticles inhibited cell proliferation in mouse xenografts, significantly suppressed BC growth and promoted chemotherapeutic sensitivity. It is most important that these nanoparticles bind strongly to tumour tissues, but not to healthy tissues.

#### 7.1.3. siRNA Based Inhibition of BC Metastases

Metastasis formation presents a problem for RNA interference and BC metastases often migrate to the lungs [224]. The exosome-coated biomimetic nanoparticles can target these metastases in animal models, and this is possible because the exosome surfaces have specific integrins binding to α6β4 and α6β1 laminin receptors on the lung surface, and the organism does not recognise these as foreign elements [224]. In particular, exosome membrane coated bio-mimetic nanoparticles have been used to inhibit *S100A4* gene. These comprise a cationine bovine serum albumin, a siS100A4 core and an exosome membrane shell. Abnormal S100A4 expression is positively associated with lung cancer tumour progression [225] and importantly its function demonstrably contributes to BC metastases migration to the lungs [226]. The nanoparticles were intravenously injected into BALB/c mice with established post-operative BC lung metastases on a daily basis for four days. This effected significant decrease in S100A4 at the protein level, accompanied by significant tumour nodule decrease after the 30-day study. It was thus confirmed that the exosome nanoparticles had great lung-targeting ability.

#### 7.1.4. Possibility of siRNA Use as a Potential Method of Improving Adoptive Transfer Therapy

Tumour-infiltrating lymphocytes (TIL) migrate from the bloodstream around and inside tumorous tissues. They are tumour-antigen specific and have a relatively high ability for solid-tumour destruction [227,228]. TIL have become important in the last twenty years in ‘adoptive transfer therapy’ where T-cells are removed from resected tissue, cultivated and re-infused in patients [229]. Their action in tumour tissues can be inhibited by immunosuppressive molecules such as PD-L1 which inhibits TIL anti-tumour activity by binding to the PD-1 receptor. The PD-1 and PD-L1 expression is rapidly elevated in later BC stages and this is associated with poor prognosis [230]. Wu et al. [231] used lipid-coated calcium phosphate nanoparticles in their inhibition of PD-1 and PD-L1. This enabled efficient siRNA entry into MCF-7 BC cell lines and subsequent inhibition of both the PD 1 receptor and ligands. The authors further reported that the elevated TIL cancer cytotoxicity was connected with increased secretion of pro-inflammatory cytokines such as IFN-γ and TNF-α. Table 5 summarises the RNA interferences performed in BC.

## 8. Conclusions

BC is one of the most common global diseases and incidence continues to rise despite long-term efforts to reduce its impact on human lives. Conventional BC treatment remains inadequate because of the heterogeneity of this disease and its high chemoresistance. The high BC frequency also effects national health budgets, and for these reasons, precise knowledge of the molecular-biological processes in these tumour cells is essential for reducing BC occurrence, improved treatment, and better course prediction.

Recent research has focused on the influence of ncRNAs’ abnormal activity in BC formation and development. These molecules regulate variable cellular processes, from individual gene transcription repression to chromatin remodelling. Moreover, the ncRNAs affect essential cellular signalling networks directly associated with tumorigenesis, and this makes them valuable potential targets for improved BC treatment. However, knowledge of their effect on BC development is still scant, and the variable functions of known ncRNAs are only now being elucidated and new ncRNAs discovered. Heightened monitoring of ncRNAs’ activity and their effect in cell lines, animal models and human tissues is therefore essential for improved BC outcomes. The resultant knowledge from this monitoring should then enhance non-invasive, liquid biopsy-based early diagnosis of this deadly disease. Finally, the precise elucidation of ncRNA activity in BC will prove beneficial in establishing a definitive molecular BC classification system.

## Figures and Tables

**Table 1:** List of lncRNAs with abnormal expression in BC cell lines, animal models and tissues.

lncRNA	Expression Rate	Target	Analysed in/Models Used in the Study
H19 [57,58,61,62,64,65,119]	Increased [95,96,100,102,103,119]	Akt signalisation [57]; precursor of miR-675 [61]; miR200/let7b [62], miR-152 [64], miR-93-5p [65], miR-675 [119]	5 BC cell lines, including paclitaxel resistant, MDA-MB-157 and MDA-MB-231 mouse xenografts [57]; 1005 healthy and 1005 BC tissues [58]; various clone cell lines, TA1 and TA2 mouse models, 108 primary and metastatic BC patients [62], MCF-7 and MDA-MB-231 cell lines, 45 healthy and tumorous tissues [64], MCF-7 and MDA-MB-231 cell lines [65], mcf-7 cell lines, plasma samples from 63 BC patients [119]
TINCR [68,70,72];	Increased [68], Increased in trastuzumab-resistant cells [70], Increased [72]	miR-7 [68], miR-125, miR 589-3 [72]	5 BC cell lines, 12 mouse xenografts, 24 BC and healthy tissues [68]; 60 BC and healthy tissues from HER2+ patients, SKBR-3 and BT474 cell lines, SKBR-3-TR and BT474-TR mouse xenografts [70], MCF-7 and MDA-MB-231 cell lines, BC and healthy tissues from 68 patients [72]
MALAT [79,80]	Increased [79,80]	miR-1 [79]; miR-204 [80]	MCF-7, MDA-MB-231 and MDA-MB-435S cell lines [79]; 4 BC cell lines, 118 BC and healthy tissues [80]
NEAT1 [83,84,85,87,88,119]	Increased [83,84,85,87,88,119]	*FOXN3* [85], miR-124 [87], miR-211 [88], miR-204 [119],	179 BC and 192 healthy tissues [83]; 40 BC and healthy tissues [84]; MCF-7 and MDA-MB-231 cell lines, mouse xenografts, 24 BC and healthy tissues [85], MCF-7, MDA-MB-231, T-47-D and ZR-75, sh-NEAT1 injected mouse models, 118 BC tissue samples [88]; MCF-7 cell lines, plasma samples from 63 BC patients [119]
DANCR [91,94,95,96]	Decreased [91], Increased [94,95,96]	EZH2 [91], EZH2 [94], miR-216a-5p [95], miR-758-3p [96]	MCF10A, MCF-7; MDA-MB-231 mouse xenografts [91] 5 BC cell lines, mouse xenografts, 46 BC and healthy tissues [94], MCF-7 and MDA-MB-23 cell lines, MDA-MB-231 mouse xenografts, 57 BC and healthy tissues [95], 4 BC cell lines, 46 BC and healthy tissues [96]
LOC554202 [99]	Decreased in luminal subtype and increased in basal subtype		Various cell lines
SNHG12 [103]		MMP13	MDA-MB-231 and BT-549cell lines, 102 BC and 95 healthy tissues
small nuclear NF90-associated lncRNA [104]	Decreased	lncRNA NRON	Hs 578T and BT-549, 70 BC and healthy tissues
BCRT1 [107]	Increased	miR-1303	5 BC cell lines, MDA-MB-231 mouse xenografts, tumorous and heathy BC tissues
HISLA [108]	Increased	PHD2 and HIF-1α	5 BC cell lines, MDA-MB-231 mouse xenografts
LINC01787 [110]	Increased	miR-125	MDA-MB-231 and MCF-7 cell lines, mouse xenografts, BC and healthy tissues from 89 patients
RP1-506.5 [111]	Increased	p27Kip	7 BC cell lines, 54 BC and healthy tissues
HOTAIR [117,119]	Increased [117,119]	miR-148 [117], miR-331 [119]	MDA-MB-231and BT549 cell lines [117], MCF-7 cell lines, plasma samples from 63 BC patients [119]

BC—Breast Cancer, lncRNA—long non-coding RNA, miR—microRNA.

**Table 2:** List of abnormally expressed piRNAs in BC cell lines and tissues.

piRNA	Expression Rate	Analyse Performed on
piRNA-651 [141]	Increased	FFPE samples of BC tumours, BCaP cell lines
piRNA-4987 [145]	Increased	54 BC and healthy tissues
piRNA-19825 [145]	Increased	54 BC and healthy tissues
piRNA-20365 [145]	Increased	54 BC and healthy tissues
piRNA-20485 [145]	Increased	54 BC and healthy tissues
piRNA-20582 [145]	Increased	54 BC and healthy tissues
piRNA-17485 [145]	Increased	54 BC and healthy tissues
piRNA-34736 [146]	Increased	MCF-7, ZR-75.1 and SKBR3 BC cells, RNAseq of 4 paired BC and healthy samples
piRNA-36249 [146]	Increased	MCF-7, ZR-75.1 and SKBR3 BC cells, RNAseq of 4 paired BC and healthy samples
piRNA-35407 [146]	Increased	MCF-7, ZR-75.1 and SKBR3 BC cells, RNAseq of 4 paired BC and healthy samples
piRNA-34377 [146]	Increased	MCF-7, ZR-75.1 and SKBR3 BC cells, RNAseq of 4 paired BC and healthy samples
piRNA-36318 [146]	Increased	MCF-7, ZR-75.1 and SKBR3 BC cells, RNAseq of 4 paired BC and healthy samples
piRNA-36026 [146]	Increased	MCF-7, ZR-75.1 and SKBR3 BC cells, RNAseq of 4 paired BC and healthy samples
piRNA-31106 [146]	Increased	MCF-7, ZR-75.1 and SKBR3 BC cells, RNAseq of 4 paired BC and healthy samples
piRNA-36743 [146]	Increased	MCF-7, ZR-75.1 and SKBR3 BC cells, RNAseq of 4 paired BC and healthy samples
piRNA-021285 [148]	Increased	441 BC and 479 healthy tissues, MCF-7 and MDA-MB-231 BC cell lines
piRNA -36712 [150]	Decreased	MCF-7 and ZR75-1, 208 BC and healthy tissues
piR-sno 75 [155]	Decreased	29 BC and healthy tissues, MCF-7 cell lines
piRNA-932 [156]	Increased	CD44 +/CD24− cells from resected tumour tissues

BC—Breast Cancer, piRNA—Piwi-interacting RNA, RNAseq—RNA sequencing, FFPE- Formalin-Fixed Paraffin-Embedded.

**Table 3:** List of snoRNA actions in BC cell lines, animal models and BC tissues.

snoRNA	Expression Rate	Target/Effect	Analyses Performed On
U15a [170]	Increased	snoRNA pathway affected p53 response	Spontaneous mouse BC
U15b [170]	Increased	snoRNA pathway affected p53 response	Spontaneous mouse BC
U22 [170]	Increased	snoRNA pathway affected p53 response	Spontaneous mouse BC
MBI-43 [170]	Increased	snoRNA pathway affected p53 response	Spontaneous mouse BC
U87 [170]	Increased	snoRNA pathway affected p53 response	Spontaneous mouse BC
HBII [170]	Increased	snoRNA pathway affected p53 response	BC tissues from resected tumours
U22 [170]	Increased	snoRNA pathway affected p53 response	BC tissues from resected tumours
U3 [170,173]	Increased	snoRNA pathway affected p53 response [170], Depletion resulted in higher p53 stability, cell cycle arrest and apoptosis [173]	BC tissues from resected tumours [170], MCF-7 cells [173]
U8 [170,173]	Increased	snoRNA pathway affected p53 response [170], Depletion resulted in higher p53 stability, cell cycle arrest and apoptosis [173]	BC tissues from resected tumours [170] MCF-7 cells [173]
U15b [170]	Increased	snoRNA pathway affected p53 response	BC tissues from resected tumours
U94 [170]	Increased	snoRNA pathway affected p53 response	BC tissues from resected tumours
U97 [170]	Increased	snoRNA pathway affected p53 response [170]	BC tissues from resected tumours
sno-miR-28 [172]	Increased	TAF9B, sno-miR-28 alters p53 protein stability through TAF9B	MDA-MB-231, 26 BC and healthy tissues
SNORD28 [172]	Increased		MDA-MB-231
SNORD25 [172]	Increased		MDA-MB-231
RNU44 [174]	Increased	Association between abnormal expression of this snoRNA and clinicopathological factors	219 BC tissues
RNU48 [174]	Increased	Association between abnormal expression of this snoRNA and clinicopathological factors	219 BC tissues
RNU43 [174]	Increased	Association between abnormal expression of this snoRNA and clinicopathological factors	219 BC tissues
RNU6B [174]	Increased	Association between abnormal expression of this snoRNA and clinicopathological factors	219 BC tissues
sdRNA-93 [174]	Increased	Regulation of *Pipox* gene, Inhibition resulted in loss of invasiveness of cell lines	MCF-7, MDA-MB-231, Luminal B Her2 + tumours

BC—Breast Cancer, snoRNA—small nucleolar RNA, snoRNA/sdRNA (small nucleolar RNAs-derived microRNAs).

**Table 4:** list of snRNA action in BC.

snRNA	Expression Rate	Target/Effect	Observed In
U1 [187]	Increased	Silencing of proximal polyadenylation signals affecting cancer cell migration and invasiveness	MCF-7, MDA-MB-231
U6 [193]	Increased	Connection with higher polymerase III activity	Human plasma samples

snRNA—small nuclear RNA.

**Table 5:** List of RNA interferences performed in BC cell lines and animal models.

siRNA	Target/Effect	Analyse Performed on	Type of Particle
siRNA-65 [211]	Inhibition of NF-κB subunit p65/decreased activity of MMP9 and cyclin D1	4T1 cell lines, mouse models with orthotopically implanted 4T1 tumours	Triple layered PEDA micelleplexes
siDANCR [214]	Lnc RNA DANCR/inhibition of DANCR resulting in PRC2-mediated H3K27-trimethylation and inhibition of Wnt/EMT signalisation	MCF-7, ZR-75, MDA-MB-231 and BT549 cell lines, MDA-MB-231 and BT549 mouse xenografts	RGD-PEG-ECO/siDANCR nanoparticles
siVEGF [217]	VEGF/inhibition of VEGF activity. Decrease of vascularisation and tumour growth	MCF-7 and MCF-7 xenografts	Vapreotide-modified core-shell nanoparticles (VAP-PLPC/siRNA VEGF NP)
BI-2536 siRNA [220]	Inhibition of PLK1 resulting in cell cycle arrest and induction of apoptosis	MDA-MB-231, Hs578T, MDA-MB-436, MDA-MB- 468 and HCC1937 cell lines	
siPLK1 [221]	Inhibition of PLK1 resulting in decrease viability of BC cell line, decreasing of *PLK1* mRNA in animal models, reduction of tumour incidence and burden in animal models	BT549 and MDA-MB-231 cell lines, Mouse models with injected LM2-4luc+/H2N cells	Mesoporous silica nanoparticle core coated layer-by-layer with bioreducible cross-linked PEI and PEG polymers, conjugated with an antibody
siPLK1 [222]	Reduction of *PLK1* mRNA resulting in decreased cell viability	MCF-7	Transfection with (HER3)-specific aptamer A30
si *EEF2* [222]	Reduction of *EEF2* mRNA resulting in decreased cell viability	MCF-7	Transfection with (HER3)-specific aptamer A30
si *GRK4* [222]	Reduction of *GRK4* mRNA resulting in decreased cell viability	MCF-7	Transfection with (HER3)-specific aptamer A30
si *SKIP5* [222]	Reduction of *SKIP5* mRNA resulting in decreased BC cell lines viability	MCF-7	Transfection with (HER3)-specific aptamer A30
XBP1 [223]	XPB1 expression decrease, resulting in lower angiogenesis and inhibited cell proliferation, significant suppression of BC growth and increased sensitivity on chemotherapy in HER2+ BC mouse mode	MDA-MB-231, MDA-MB-453, MCF-7 and BT474 cell lines, BT474 mouse xenografts	RNase resistant RNA nanoparticle with specific aptamers (3WJ-HER2apt-siXBP1)
S100A4 [225]	Decrease of S100A4 at the protein level, decrease of tumour nodules after 30 days	BALB/c mice with inoculated 4T1 cells	Cationic bovine serum albumin conjugated siS100A4 and exosome membrane coated nanoparticles
PD-1/PD-1 ligands [231]	Inhibition of PD-1 receptor and ligand activity, increase of inflammatory cytokines, increase of tumour infiltrating lymphocytes’ killing efficiency	MCF-7	Lipid-modified calcium phosphate nanoparticles round-shaped with positively charged surface.

BC—Breast Cancer, siRNA—short interfering RNA.

## Abbreviations

Aub	Aubergine
BC	Breast cancer
ceRNA	Competiting endogenous RNA
circRNA	Circular RNA
ciRNA	Circular intronic RNA
DCIS	Ductal carcinoma in-situ
DNMT1	DNA methyltransferase 1
EMT	Epithelial-to- mesenchymal transition
ER	Oestrogen receptor
EV	Extracellular vesicle
FBL	Fibrilarin
GEDPR	G-protein-coupled oestrogen receptors
HER2	Human epidermal growth factor receptor 2
HIF-1α	Hypoxia inducible factor-1a
LCIS	Lobular carcinoma in-situ
lncRNA	Long non-coding RNA
miR	MicroRNA
miRNA	MicroRNA
MRE	MicroRNA response elements
NAT	Natural antisense transcripts
ncRNA	Non-coding RNA
NST	Invasive carcinoma of no special type
PAS	Proximal polyadenylation signals
piRNA	Piwi-interacting RNA
PR	Progesterone receptor
pre-miRNA	Precursor microRNA
pre-shRNA	Precursor short hairpin RNA
pri-miRNA	Primary microRNA
pri-shRNA	Primary short hairpin RNA
PTGS	Post-transcriptional gene silencing
RISC	RNA-induced silencing complex
RNAi	RNA interference
rRNA	Ribosomal RNA
sdRNA	Small nucleolar RNAs-derived microRNAs
shRNA	Short hairpin RNA
siRNA	Small interfering RNA
snaR	Small nuclear NF90-associated lncRNA
sno-miRNA	Small nucleolar RNAs-derived microRNAs’
snoRNA	Small nucleolar RNA
snRNA	Small nuclear RNA
SPA	5′ snoRNA-ended and 3′-polyadenylated lncRNA
TAM	Tumour associated macrophages
TE	Transposable elements
TERC	Telomerase RNA
TGS	Transcriptional gene silencing
TIL	Tumour-infiltrating lymphocytes
TNBC	Triple negative breast cancer
tRNA	Transfer RNA

## References

[1] Ferlay J., Colombet M., Soerjomataram I., Mathers C., Parkin D.M., Piñeros M., Znaor A., Bray F. (2019). Estimating the Global Cancer Incidence and Mortality in 2018: GLOBOCAN Sources and Methods. Int. J. Cancer.

[2] Bray F., Ferlay J., Laversanne M., Brewster D.H., Gombe Mbalawa C., Kohler B., Piñeros M., Steliarova-Foucher E., Swaminathan R., Antoni S. (2015). Cancer Incidence in Five Continents: Inclusion Criteria, Highlights from Volume X and the Global Status of Cancer Registration. Int. J. Cancer.

[3] Torre L.A., Siegel R.L., Ward E.M., Jemal A. (2016). Global Cancer Incidence and Mortality Rates and Trends—An Update. Cancer Epidemiol. Biomark. Prev..

[4] Dafni U., Tsourti Z., Alatsathianos I. (2019). Breast Cancer Statistics in the European Union: Incidence and Survival across European Countries. Breast Care..

[5] Ng C.J., Teo C.H., Abdullah N., Tan W.P., Tan H.M. (2015). Relationships between Cancer Pattern, Country Income and Geographical Region in Asia. BMC Cancer.

[6] Adeloye D., Sowunmi O.Y., Jacobs W., David R.A., Adeosun A.A., Amuta A.O., Misra S., Gadanya M., Auta A., Harhay M.O. (2018). Estimating the Incidence of Breast Cancer in Africa: A Systematic Review and Meta-Analysis. J. Glob. Health.

[7] Winters S., Martin C., Murphy D., Shokar N.K. (2017). Breast Cancer Epidemiology, Prevention, and Screening. Prog. Mol. Biol. Transl. Sci..

[8] Kohler B.A., Sherman R.L., Howlader N., Jemal A., Ryerson A.B., Henry K.A., Boscoe F.P., Cronin K.A., Lake A., Noone A.M. (2015). Annual Report to the Nation on the Status of Cancer, 1975–2011, Featuring Incidence of Breast Cancer Subtypes by Race/Ethnicity, Poverty, and State. J. Natl. Cancer Inst..

[9] Harbeck N., Penault-Llorca F., Cortes J., Gnant M., Houssami N., Poortmans P., Ruddy K., Tsang J., Cardoso F. (2019). Breast Cancer. Nat. Rev. Dis. Prim..

[10] Eliyatkin N., Yalcin E., Zengel B., Aktaş S., Vardar E. (2015). Molecular Classification of Breast Carcinoma: From Traditional, Old-Fashioned Way to A New Age, and A New Way. J. Breast Health.

[11] Sinn H.P., Kreipe H. (2013). A Brief Overview of the WHO Classification of Breast Tumors. Breast Care.

[12] Ward E.M., DeSantis C.E., Lin C.C., Kramer J.L., Jemal A., Kohler B., Brawley O.W., Gansler T. (2015). Cancer Statistics: Breast Cancer in Situ. CA Cancer J. Clin..

[13] Sørlie T., Perou C.M., Tibshirani R., Aas T., Geisler S., Johnsen H., Hastie T., Eisen M.B., Van De Rijn M., Jeffrey S.S. (2001). Gene Expression Patterns of Breast Carcinomas Distinguish Tumor Subclasses with Clinical Implications. Proc. Natl. Acad. Sci. USA.

[14] Hu Z., Fan C., Oh D.S., Marron J.S., He X., Qaqish B.F., Livasy C., Carey L.A., Reynolds E., Dressler L. (2006). The Molecular Portraits of Breast Tumors Are Conserved across Microarray Platforms. BMC Genom..

[15] Bernard P.S., Parker J.S., Mullins M., Cheung M.C.U., Leung S., Voduc D., Vickery T., Davies S., Fauron C., He X. (2009). Supervised Risk Predictor of Breast Cancer Based on Intrinsic Subtypes. J. Clin. Oncol..

[16] Fougner C., Bergholtz H., Norum J.H., Sørlie T. (2020). Re-Definition of Claudin-Low as a Breast Cancer Phenotype. Nat. Commun..

[17] Zhang J., Abrams Z., Parvin J.D., Huang K. (2016). Integrative Analysis of Somatic Mutations and Transcriptomic Data to Functionally Stratify Breast Cancer Patients. BMC Genom..

[18] Suo C., Hrydziuszko O., Lee D., Pramana S., Saputra D., Joshi H., Calza S., Pawitan Y. (2015). Integration of Somatic Mutation, Expression and Functional Data Reveals Potential Driver Genes Predictive of Breast Cancer Survival. Bioinformatics.

[19] Shlien A., Raine K., Fuligni F., Arnold R., Nik-Zainal S., Dronov S., Mamanova L., Rosic A., Ju Y.S., Cooke S.L. (2016). Direct transcriptional consequences of somatic mutation in breast cancer. Cell Rep..

[20] Dai X., Li T., Bai Z., Yang Y., Liu X., Zhan J., Shi B. (2015). Breast Cancer Intrinsic Subtype Classification, Clinical Use and Future Trends. Am. J. Cancer Res..

[21] Waks A.G., Winer E.P. (2019). Breast Cancer Treatment: A Review. JAMA.

[22] Sole C., Arnaiz E., Manterola L., Otaegui D., Lawrie C.H. (2019). The Circulating Transcriptome as a Source of Cancer Liquid Biopsy Biomarkers. Semin. Cancer Biol..

[23] Djebali S., Davis C.A., Merkel A., Dobin A., Lassmann T., Mortazavi A., Tanzer A., Lagarde J., Lin W., Schlesinger F. (2012). Landscape of Transcription in Human Cells. Nature.

[24] Huang T., Alvarez A., Hu B., Cheng S.Y. (2013). Noncoding RNAs in Cancer and Cancer Stem Cells. Chin. J. Cancer.

[25] Wang W.T., Han C., Sun Y.M., Chen T.Q., Chen Y.Q. (2019). Noncoding RNAs in Cancer Therapy Resistance and Targeted Drug Development. J. Hematol. Oncol..

[26] Yao R.W., Wang Y., Chen L.L. (2019). Cellular Functions of Long Noncoding RNAs. Nat. Cell Biol..

[27] Adams B.D., Parsons C., Walker L., Zhang W.C., Slack F.J. (2017). Targeting Noncoding RNAs in Disease. J. Clin. Investig..

[28] Kanekura K., Nishi H., Isaka K., Kuroda M. (2016). MicroRNA and Gynecologic Cancers. J. Obstet. Gynaecol. Res..

[29] Liolios T., Kastora S.L., Colombo G. (2019). MicroRNAs in Female Malignancies. Cancer Inform..

[30] Gebert L.F.R., MacRae I.J. (2019). Regulation of MicroRNA Function in Animals. Nat. Rev. Mol. Cell Biol..

[31] O’Brien J., Hayder H., Zayed Y., Peng C. (2018). Overview of MicroRNA Biogenesis, Mechanisms of Actions, and Circulation. Front. Endocrinol..

[32] Tanzer A., Stadler P.F. (2004). Molecular Evolution of a MicroRNA Cluster. J. Mol. Biol..

[33] Bartel D.P. (2009). MicroRNAs: Target Recognition and Regulatory Functions. Cell.

[34] Kozomara A., Griffiths-Jones S. (2014). MiRBase: Annotating High Confidence MicroRNAs Using Deep Sequencing Data. Nucleic Acids Res..

[35] Denli A.M., Tops B.B.J., Plasterk R.H.A., Ketting R.F., Hannon G.J. (2004). Processing of Primary MicroRNAs by the Microprocessor Complex. Nature.

[36] Broughton J.P., Lovci M.T., Huang J.L., Yeo G.W., Pasquinelli A.E. (2016). Pairing beyond the Seed Supports MicroRNA Targeting Specificity. Mol. Cell.

[37] Bukhari S.I.A., Truesdell S.S., Lee S., Kollu S., Classon A., Boukhali M., Jain E., Mortensen R.D., Yanagiya A., Sadreyev R.I. (2016). A Specialized Mechanism of Translation Mediated by FXR1a-Associated MicroRNP in Cellular Quiescence. Mol. Cell.

[38] Truesdell S.S., Mortensen R.D., Seo M., Schroeder J.C., Lee J.H., Letonqueze O., Vasudevan S.V. (2012). MicroRNA-Mediated MRNA Translation Activation in Quiescent Cells and Oocytes Involves Recruitment of a Nuclear MicroRNP. Sci. Rep..

[39] Peng Y., Croce C.M. (2016). The Role of MicroRNAs in Human Cancer. Signal Transduct. Target. Ther..

[40] Ma L., Cao J., Liu L., Du Q., Li Z., Zou D., Bajic V.B., Zhang Z. (2019). Lncbook: A Curated Knowledgebase of Human Long Non-Coding Rnas. Nucleic Acids Res..

[41] Wang K.C., Chang H.Y. (2011). Molecular Mechanisms of Long Noncoding RNAs. Mol. Cell.

[42] Shukla C.J., McCorkindale A.L., Gerhardinger C., Korthauer K.D., Cabili M.N., Shechner D.M., Irizarry R.A., Maass P.G., Rinn J.L. (2018). High-throughput Identification of RNA Nuclear Enrichment Sequences. EMBO J..

[43] Zhang B., Gunawardane L., Niazi F., Jahanbani F., Chen X., Valadkhan S. (2014). A Novel RNA Motif Mediates the Strict Nuclear Localization of a Long Noncoding RNA. Mol. Cell. Biol..

[44] Hall L.L., Lawrence J.B. (2010). XIST RNA and Architecture of the Inactive X Chromosome Implications for the Repeat Genome. Cold Spring Harb. Symp. Quant. Biol..

[45] Hall L.L., Carone D.M., Gomez A.V., Kolpa H.J., Byron M., Mehta N., Fackelmayer F.O., Lawrence J.B. (2014). Stable C0T-1 Repeat RNA Is Abundant and Is Associated with Euchromatic Interphase Chromosomes. Cell.

[46] Zhao J., Sun B.K., Erwin J.A., Song J.J., Lee J.T. (2008). Polycomb Proteins Targeted by a Short Repeat RNA to the Mouse X Chromosome. Science.

[47] Han P., Li W., Lin C.H., Yang J., Shang C., Nurnberg S.T., Jin K.K., Xu W., Lin C.Y., Lin C.J. (2014). A Long Noncoding RNA Protects the Heart from Pathological Hypertrophy. Nature.

[48] Tang Y., Wang J., Lian Y., Fan C., Zhang P., Wu Y., Li X., Xiong F., Li X., Li G. (2017). Linking Long Non-Coding RNAs and SWI/SNF Complexes to Chromatin Remodeling in Cancer. Mol. Cancer.

[49] Terashima M., Tange S., Ishimura A., Suzuki T. (2017). MEG3 Long Noncoding RNA Contributes to the Epigenetic Regulation of Epithelial-Mesenchymal Transition in Lung Cancer Cell Lines. J. Biol. Chem..

[50] Jain A.K., Xi Y., McCarthy R., Allton K., Akdemir K.C., Patel L.R., Aronow B., Lin C., Li W., Yang L. (2016). LncPRESS1 Is a P53-Regulated LncRNA That Safeguards Pluripotency by Disrupting SIRT6-Mediated De-Acetylation of Histone H3K56. Mol. Cell.

[51] Arab K., Park Y.J., Lindroth A.M., Schäfer A., Oakes C., Weichenhan D., Lukanova A., Lundin E., Risch A., Meister M. (2014). Long Noncoding RNA TARID Directs Demethylation and Activation of the Tumor Suppressor TCF21 via GADD45A. Mol. Cell.

[52] Deng J., Mueller M., Geng T., Shen Y., Liu Y., Hou P., Ramillapalli R., Taylor H.S., Paidas M., Huang Y. (2017). H19 LncRNA Alters Methylation and Expression of Hnf4α in the Liver of Metformin-Exposed Fetuses. Cell Death Dis..

[53] Derrien T., Guigó R., Johnson R. (2012). The Long Non-Coding Rnas: A New (p)Layer in the “Dark Matter”. Front. Genet..

[54] Mercer T.R., Dinger M.E., Mattick J.S. (2009). Long Non-Coding RNAs: Insights into Functions. Nat. Rev. Genet..

[55] Tam C., Wong J.H., Tsui S.K.W., Zuo T., Chan T.F., Ng T.B. (2019). LncRNAs with MiRNAs in Regulation of Gastric, Liver, and Colorectal Cancers: Updates in Recent Years. Appl. Microbiol. Biotechnol..

[56] Wang J., Sun J., Yang F. (2020). The Role of Long Non-coding RNA H19 in Breast Cancer (Review). Oncol. Lett..

[57] Han J., Han B., Wu X., Hao J., Dong X., Shen Q., Pang H. (2018). Knockdown of LncRNA H19 Restores Chemo-Sensitivity in Paclitaxel-Resistant Triple-Negative Breast Cancer through Triggering Apoptosis and Regulating Akt Signaling Pathway. Toxicol. Appl. Pharmacol..

[58] Lin Y., Fu F., Chen Y., Qiu W., Lin S., Yang P., Huang M., Wang C. (2017). Genetic Variants in Long Noncoding RNA H19 Contribute to the Risk of Breast Cancer in a Southeast China Han Population. Onco. Targets Ther..

[59] Cui P., Zhao Y., Chu X., He N., Zheng H., Han J., Song F., Chen K. (2018). SNP Rs2071095 in LincRNA H19 Is Associated with Breast Cancer Risk. Breast Cancer Res. Treat..

[60] Berteaux N., Lottin S., Monté D., Pinte S., Quatannens B., Coll J., Hondermarck H., Curgy J.J., Dugimont T., Adriaenssens E. (2005). H19 MRNA-like Noncoding RNA Promotes Breast Cancer Cell Proliferation through Positive Control by E2F1. J. Biol. Chem..

[61] Collette J., Le Bourhis X., Adriaenssens E. (2017). Regulation of Human Breast Cancer by the Long Non-Coding RNA H19. Int. J. Mol. Sci..

[62] Zhou W., Ye X.L., Xu J., Cao M.G., Fang Z.Y., Li L.Y., Guan G.H., Liu Q., Qian Y.H., Xie D. (2017). The LncRNA H19 Mediates Breast Cancer Cell Plasticity during EMT and MET Plasticity by Differentially Sponging MiR-200b/c and Let-7b. Sci. Signal..

[63] Sánchez-Cid L., Pons M., Lozano J.J., Rubio N., Guerra-Rebollo M., Soriano A., Paris-Coderch L., Segura M.F., Fueyo R., Arguimbau J. (2017). MicroRNA-200, Associated with Metastatic Breast Cancer, Promotes Traits of Mammary Luminal Progenitor Cells. Oncotarget.

[64] Li Z., Li Y., Li Y., Ren K., Li X., Han X., Wang J. (2017). Long Non-Coding RNA H19 Promotes the Proliferation and Invasion of Breast Cancer through Upregulating DNMT1 Expression by Sponging MiR-152. J. Biochem. Mol. Toxicol..

[65] Li J.P., Xiang Y., Fan L.J., Yao A., Li H., Liao X.H. (2019). Long Noncoding RNA H19 Competitively Binds MiR-93-5p to Regulate STAT3 Expression in Breast Cancer. J. Cell. Biochem..

[66] Barsyte-Lovejoy D., Lau S.K., Boutros P.C., Khosravi F., Jurisica I., Andrulis I.L., Tsao M.S., Penn L.Z. (2006). The C-Myc Oncogene Directly Induces the H19 Noncoding RNA by Allele-Specific Binding to Potentiate Tumorigenesis. Cancer Res..

[67] Popkie A.P., Zeidner L.C., Albrecht A.M., D’Ippolito A., Eckardt S., Newsom D.E., Groden J., Doble B.W., Aronow B., McLaughlin K.J. (2010). Phosphatidylinositol 3-Kinase (PI3K) Signaling via Glycogen Synthase Kinase-3 (Gsk-3) Regulates DNA Methylation of Imprinted Loci. J. Biol. Chem..

[68] Liu Y., Du Y., Hu X., Zhao L., Xia W. (2018). Up-Regulation of CeRNA TINCR by SP1 Contributes to Tumorigenesis in Breast Cancer. BMC Cancer.

[69] Okuda H., Xing F., Pandey P.R., Sharma S., Watabe M., Pai S.K., Mo Y.Y., Iiizumi-Gairani M., Hirota S., Liu Y. (2013). MiR-7 Suppresses Brain Metastasis of Breast Cancer Stem-like Cells by Modulating KLF4. Cancer Res..

[70] Dong H., Hu J., Zou K., Ye M., Chen Y., Wu C., Chen X., Han M. (2019). Activation of LncRNA TINCR by H3K27 Acetylation Promotes Trastuzumab Resistance and Epithelial-Mesenchymal Transition by Targeting MicroRNA-125b in Breast Cancer 11 Medical and Health Sciences 1112 Oncology and Carcinogenesis. Mol. Cancer.

[71] Ferracin M., Bassi C., Pedriali M., Pagotto S., D’Abundo L., Zagatti B., Corrà F., Musa G., Callegari E., Lupini L. (2013). MiR-125b Targets Erythropoietin and Its Receptor and Their Expression Correlates with Metastatic Potential and ERBB2/HER2 Expression. Mol. Cancer.

[72] Guo F., Zhu X., Zhao Q., Huang Q. (2020). MiR-589-3p Sponged by the LncRNA TINCR Inhibits the Proliferation, Migration and Invasion and Promotes the Apoptosis of Breast Cancer Cells by Suppressing the Akt Pathway via IGF1R. Int. J. Mol. Med..

[73] Simpson A., Petnga W., Macaulay V.M., Weyer-Czernilofsky U., Bogenrieder T. (2017). Insulin-Like Growth Factor (IGF) Pathway Targeting in Cancer: Role of the IGF Axis and Opportunities for Future Combination Studies. Target. Oncol..

[74] Riedemann J., Macaulay V.M. (2006). IGF1R Signalling and Its Inhibition. Endocr. Relat. Cancer.

[75] Wu Y., Shao A., Wang L., Hu K., Yu C., Pan C., Zhang S. (2019). The Role of LncRNAs in the Distant Metastasis of Breast Cancer. Front. Oncol..

[76] Jadaliha M., Zong X., Malakar P., Ray T., Singh D.K., Freier S.M., Jensen T., Prasanth S.G., Karni R., Ray P.S. (2016). Functional and Prognostic Significance of Long Non-Coding RNA MALAT1 as a Metastasis Driver in ER Negative Lymph Node Negative Breast Cancer. Oncotarget.

[77] Zhao Z., Chen C., Liu Y., Wu C. (2014). 17β-Estradiol Treatment Inhibits Breast Cell Proliferation, Migration and Invasion by Decreasing MALAT-1 RNA Level. Biochem. Biophys. Res. Commun..

[78] Li Z., Xu L., Liu Y., Fu S., Tu J., Hu Y., Xiong Q. (2018). LncRNA MALAT1 Promotes Relapse of Breast Cancer Patients with Postoperative Fever. Am. J. Transl. Res..

[79] Chou J., Wang B., Zheng T., Li X., Zheng L., Hu J., Zhang Y., Xing Y., Xi T. (2016). MALAT1 Induced Migration and Invasion of Human Breast Cancer Cells by Competitively Binding MIR-1 with Cdc42. Biochem. Biophys. Res. Commun..

[80] Wang Y., Zhou Y., Yang Z., Chen B., Huang W., Liu Y., Zhang Y. (2017). MiR-204/ZEB2 Axis Functions as Key Mediator for MALATI-Induced Epithelial-Mesenchymal Transition in Breast Cancer. Tumor Biol..

[81] Vandewalle C., Comijn J., De Craene B., Vermassen P., Bruyneel E., Andersen H., Tulchinsky E., Van Roy F., Berx G. (2005). SIP1/ZEB2 Induces EMT by Repressing Genes of Different Epithelial Cell-Cell Junctions. Nucleic Acids Res..

[82] Lu Y., Li T., Wei G., Liu L., Chen Q., Xu L., Zhang K., Zeng D., Liao R. (2016). The Long Non-Coding RNA NEAT1 Regulates Epithelial to Mesenchymal Transition and Radioresistance in through MiR-204/ZEB1 Axis in Nasopharyngeal Carcinoma. Tumor Biol..

[83] Shin V.Y., Chen J., Cheuk I.W.Y., Siu M.T., Ho C.W., Wang X., Jin H., Kwong A. (2019). Long Non-Coding RNA NEAT1 Confers Oncogenic Role in Triple-Negative Breast Cancer through Modulating Chemoresistance and Cancer Stemness. Cell Death Dis..

[84] Zhang M., Wu W.B., Wang Z.W., Wang X.H. (2017). LncRNA NEAT1 Is Closely Related with Progression of Breast Cancer via Promoting Proliferation and EMT. Eur. Rev. Med. Pharmacol. Sci..

[85] Li W., Zhang Z., Liu X., Cheng X., Zhang Y., Han X., Zhang Y., Liu S., Yang J., Xu B. (2017). The FOXN3-NEAT1-SIN3A Repressor Complex Promotes Progression of Hormonally Responsive Breast Cancer. J. Clin. Investig..

[86] Yan W., Cao Q.J., Arenas R.B., Bentley B., Shao R. (2010). GATA3 Inhibits Breast Cancer Metastasis through the Reversal of Epithelial-Mesenchymal Transition. J. Biol. Chem..

[87] Ma J.H., Qin L., Li X. (2020). Role of STAT3 Signaling Pathway in Breast Cancer. Cell Commun. Signal..

[88] Li X., Wang S., Li Z., Long X., Guo Z., Zhang G., Zu J., Chen Y., Wen L. (2017). The LncRNA NEAT1 Facilitates Cell Growth and Invasion via the MiR-211/HMGA2 Axis in Breast Cancer. Int. J. Biol. Macromol..

[89] Pei Y., Yao Q., Li Y., Zhang X., Xie B. (2019). MicroRNA-211 Regulates Cell Proliferation, Apoptosis and Migration/Invasion in Human Osteosarcoma via Targeting EZRIN. Cell. Mol. Biol. Lett..

[90] Qu X., Gao D., Ren Q., Jiang X., Bai J., Sheng L. (2018). MiR-211 Inhibits Proliferation, Invasion and Migration of Cervical Cancer via Targeting SPARC. Oncol. Lett..

[91] Li Z., Hou P., Fan D., Dong M., Ma M., Li H., Yao R., Li Y., Wang G., Geng P. (2017). The Degradation of EZH2 Mediated by LncRNA ANCR Attenuated the Invasion and Metastasis of Breast Cancer. Cell Death Differ..

[92] Li L., Liu J., Xue H., Li C., Liu Q., Zhou Y., Wang T., Wang H., Qian H., Wen T. (2020). A TGF-β-MTA1-SOX4-EZH2 Signaling Axis Drives Epithelial–Mesenchymal Transition in Tumor Metastasis. Oncogene.

[93] Kim K.H., Roberts C.W.M. (2016). Targeting EZH2 in Cancer. Nat. Med..

[94] Zhang K., Tan X., Guo L. (2020). The Long Non-coding RNA DANCR Regulates the Inflammatory Phenotype of Breast Cancer Cells and Promotes Breast Cancer Progression *via* EZH2-dependent Suppression of SOCS3 Transcription. Mol. Oncol..

[95] Tao W., Wang C., Zhu B., Zhang G., Pang D. (2019). LncRNA DANCR Contributes to Tumor Progression via Targetting MiR-216a-5p in Breast Cancer: LncRNA DANCR Contributes to Tumor Progression. Biosci. Rep..

[96] Zhang X.H., Li B.F., Ding J., Shi L., Ren H.M., Liu K., Huang C.C., Ma F.X., Wu X.Y. (2020). Lncrna Dancr-Mir-758-3p-Pax6 Molecular Network Regulates Apoptosis and Autophagy of Breast Cancer Cells. Cancer Manag. Res..

[97] Xia X., Yin W., Zhang X., Yu X., Wang C., Xu S., Feng W., Yang H. (2015). PAX6 Overexpression Is Associated with the Poor Prognosis of Invasive Ductal Breast Cancer. Oncol. Lett..

[98] Geyer F.C., Pareja F., Weigelt B., Rakha E., Ellis I.O., Schnitt S.J., Reis-Filho J.S. (2017). The Spectrum of Triple-Negative Breast Disease: High- and Low-Grade Lesions. Am. J. Pathol..

[99] Augoff K., McCue B., Plow E.F., Sossey-Alaoui K. (2012). MiR-31 and Its Host Gene LncRNA LOC554202 Are Regulated by Promoter Hypermethylation in Triple-Negative Breast Cancer. Mol. Cancer.

[100] Luo L.J., Yang F., Ding J.J., Yan D.L., Wang D.D., Yang S.J., Ding L., Li J., Chen D., Ma R. (2016). MiR-31 Inhibits Migration and Invasion by Targeting SATB2 in Triple Negative Breast Cancer. Gene.

[101] Koduru S.V., Tiwari A.K., Leberfinger A., Hazard S.W., Kawasawa Y.I., Mahajan M., Ravnic D.J. (2017). A Comprehensive NGS Data Analysis of Differentially Regulated MiRNAs, PiRNAs, LncRNas and Sn/SnoRNAs in Triple Negative Breast Cancer. J. Cancer.

[102] Zimta A.A., Tigu A.B., Braicu C., Stefan C., Ionescu C., Berindan-Neagoe I. (2020). An Emerging Class of Long Non-Coding RNA With Oncogenic Role Arises From the SnoRNA Host Genes. Front. Oncol..

[103] Wang O.C., Yang F., Liu Y.H., Lv L., Ma R., Chen C., Wang J., Tan Q., Cheng Y., Xia E. (2017). C-MYC-Induced Upregulation of LncRNA SNHG12 Regulates Cell Proliferation, Apoptosis and Migration in Triple-Negative Breast Cancer. Am. J. Transl. Res..

[104] Leeman M.F., Curran S., Murray G.I. (2002). The Structure, Regulation, and Function of Human Matrix Metalloproteinase-13. Critical Rev. Biochem. Mol. Biol..

[105] Lee J., Jung J.H., Chae Y.S., Park H.Y., Kim W.W., Lee S.J., Jeong J.H., Kang S.H. (2016). Long Noncoding RNA SnaR Regulates Proliferation, Migration and Invasion of Triple-Negative Breast Cancer Cells. Anticancer Res..

[106] Niu L., Fan Q., Yan M., Wang L. (2019). LncRNA NRON Down-Regulates LncRNA SnaR and Inhibits Cancer Cell Proliferation in TNBC. Biosci. Rep..

[107] Liang Y., Song X., Li Y., Chen B., Zhao W., Wang L., Zhang H., Liu Y., Han D., Zhang N. (2020). LncRNA BCRT1 Promotes Breast Cancer Progression by Targeting MiR-1303/PTBP3 Axis. Mol. Cancer.

[108] Chen F., Chen J., Yang L., Liu J., Zhang X., Zhang Y., Tu Q., Yin D., Lin D., Wong P.P. (2019). Extracellular Vesicle-Packaged HIF-1α-Stabilizing LncRNA from Tumour-Associated Macrophages Regulates Aerobic Glycolysis of Breast Cancer Cells. Nat. Cell Biol..

[109] Hsu P.P., Sabatini D.M. (2008). Cancer Cell Metabolism: Warburg and Beyond. Cell.

[110] Li Y., Song Y., Wang Z., Zhang Z., Lu M., Wang Y. (2019). Long Non-Coding RNA LINC01787 Drives Breast Cancer Progression via Disrupting MiR-125b Generation. Front. Oncol..

[111] Jia X., Shi L., Wang X., Luo L., Ling L., Yin J., Song Y., Zhang Z., Qiu N., Liu H. (2019). KLF5 Regulated LncRNA RP1 Promotes the Growth and Metastasis of Breast Cancer via Repressing P27kip1 Translation. Cell Death Dis..

[112] Jia L., Zhou Z., Liang H., Wu J., Shi P., Li F., Wang Z., Wang C., Chen W., Zhang H. (2016). KLF5 Promotes Breast Cancer Proliferation, Migration and Invasion in Part by Upregulating the Transcription of TNFAIP2. Oncogene.

[113] Fagoonee S., Durazzo M. (2017). HOTAIR and Gastric Cancer: A Lesson from Two Meta-Analyses. Panminerva Med..

[114] Zhou Y., Wang C., Liu X., Wu C., Yin H. (2017). Long Non-Coding RNA HOTAIR Enhances Radioresistance in MDA-MB231 Breast Cancer Cells. Oncol. Lett..

[115] Sørensen K.P., Thomassen M., Tan Q., Bak M., Cold S., Burton M., Larsen M.J., Kruse T.A. (2013). Long Non-Coding RNA HOTAIR Is an Independent Prognostic Marker of Metastasis in Estrogen Receptor-Positive Primary Breast Cancer. Breast Cancer Res. Treat..

[116] Bhan A., Hussain I., Ansari K.I., Kasiri S., Bashyal A., Mandal S.S. (2013). Antisense Transcript Long Noncoding RNA (LncRNA) HOTAIR Is Transcriptionally Induced by Estradiol. J. Mol. Biol..

[117] Tao S., He H., Chen Q. (2015). Estradiol Induces HOTAIR Levels via GPER-Mediated MiR-148a Inhibition in Breast Cancer. J. Transl. Med..

[118] Xu Q., Jiang Y., Yin Y., Li Q., He J., Jing Y., Qi Y.T., Xu Q., Li W., Lu B. (2013). A Regulatory Circuit of MiR-148a/152 and DNMT1 in Modulating Cell Transformation and Tumor Angiogenesis through IGF-IR and IRS1. J. Mol. Cell Biol..

[119] Müller V., Oliveira-Ferrer L., Steinbach B., Pantel K., Schwarzenbach H. (2019). Interplay of LncRNA H19/MiR-675 and LncRNA NEAT1/MiR-204 in Breast Cancer. Mol. Oncol..

[120] Maleki Dana P., Mansournia M.A., Mirhashemi S.M. (2020). PIWI-Interacting RNAs: New Biomarkers for Diagnosis and Treatment of Breast Cancer. Cell Biosci..

[121] Ishizu H., Siomi H., Siomi M.C. (2012). Biology of Piwi-Interacting RNAs: New Insights into Biogenesis and Function inside and Outside of Germlines. Genes Dev..

[122] Yu Y., Xiao J., Hann S.S. (2019). The Emerging Roles of PIWI-Interacting RNA in Human Cancers. Cancer Manag. Res..

[123] Martinez V.D., Vucic E.A., Thu K.L., Hubaux R., Enfield K.S.S., Pikor L.A., Becker-Santos D.D., Brown C.J., Lam S., Lam W.L. (2015). Unique Somatic and Malignant Expression Patterns Implicate PIWI-Interacting RNAs in Cancer-Type Specific Biology. Sci. Rep..

[124] Czech B., Munafò M., Ciabrelli F., Eastwood E.L., Fabry M.H., Kneuss E., Hannon G.J. (2018). PiRNA-Guided Genome Defense: From Biogenesis to Silencing. Annu. Rev. Genet..

[125] Aravin A.A., Sachidanandam R., Bourc’his D., Schaefer C., Pezic D., Toth K.F., Bestor T., Hannon G.J. (2008). A PiRNA Pathway Primed by Individual Transposons Is Linked to De Novo DNA Methylation in Mice. Mol. Cell.

[126] Dai P., Wang X., Liu M.F. (2020). A Dual Role of the PIWI/PiRNA Machinery in Regulating MRNAs during Mouse Spermiogenesis. Sci. China Life Sci..

[127] Peng J.C., Lin H. (2013). Beyond Transposons: The Epigenetic and Somatic Functions of the Piwi-PiRNA Mechanism. Curr. Opin. Cell Biol..

[128] Rajasethupathy P., Antonov I., Sheridan R., Frey S., Sander C., Tuschl T., Kandel E.R. (2012). A Role for Neuronal PiRNAs in the Epigenetic Control of Memory-Related Synaptic Plasticity. Cell.

[129] Lee E.J., Banerjee S., Zhou H., Jammalamadaka A., Arcila M., Manjunath B.S., Kosik K.S. (2011). Identification of PiRNAs in the Central Nervous System. RNA.

[130] Rouget C., Papin C., Boureux A., Meunier A.C., Franco B., Robine N., Lai E.C., Pelisson A., Simonelig M. (2010). Maternal MRNA Deadenylation and Decay by the PiRNA Pathway in the Early Drosophila Embryo. Nature.

[131] Luteijn M.J., Ketting R.F. (2013). PIWI-Interacting RNAs: From Generation to Transgenerational Epigenetics. Nat. Rev. Genet..

[132] Watanabe T., Lin H. (2014). Posttranscriptional Regulation of Gene Expression by Piwi Proteins and PiRNAs. Mol. Cell.

[133] Krishnan P., Damaraju S. (2018). The Challenges and Opportunities in the Clinical Application of Noncoding RNAs: The Road Map for MiRNAs and PiRNAs in Cancer Diagnostics and Prognostics. Int. J. Genom..

[134] Weng W., Li H., Goel A. (2019). Piwi-Interacting RNAs (PiRNAs) and Cancer: Emerging Biological Concepts and Potential Clinical Implications. Biochim. Biophys. Acta Rev. Cancer.

[135] Ng K.W., Anderson C., Marshall E.A., Minatel B.C., Enfield K.S.S., Saprunoff H.L., Lam W.L., Martinez V.D. (2016). Piwi-Interacting RNAs in Cancer: Emerging Functions and Clinical Utility. Mol. Cancer.

[136] Siddiqi S., Matushansky I. (2012). Piwis and Piwi-Interacting RNAs in the Epigenetics of Cancer. J. Cell. Biochem..

[137] Lu Y., Li C., Zhang K., Sun H., Tao D., Liu Y., Zhang S., Ma Y. (2010). Identification of PiRNAs in Hela Cells by Massive Parallel Sequencing. BMB Rep..

[138] Fathizadeh H., Asemi Z. (2019). Epigenetic Roles of PIWI Proteins and PiRNAs in Lung Cancer. Cell Biosci..

[139] Peng L., Song L., Liu C., Lv X., Li X., Jie J., Zhao D., Li D. (2016). PiR-55490 Inhibits the Growth of Lung Carcinoma by Suppressing MTOR Signaling. Tumor Biol..

[140] Sellitto A., Geles K., D’Agostino Y., Conte M., Alexandrova E., Rocco D., Nassa G., Giurato G., Tarallo R., Weisz A. (2019). Molecular and Functional Characterization of the Somatic PIWIL1/PiRNA Pathway in Colorectal Cancer Cells. Cells.

[141] Cheng J., Guo J.M., Xiao B.X., Miao Y., Jiang Z., Zhou H., Li Q.N. (2011). PiRNA, the New Non-Coding RNA, Is Aberrantly Expressed in Human Cancer Cells. Clin. Chim. Acta.

[142] Yao J., Wang Y.W., Fang B.B., Zhang S.J., Cheng B.L. (2016). PiR-651 and Its Function in 95-D Lung Cancer Cells. Biomed. Rep..

[143] Li D., Luo Y., Gao Y., Yang Y., Wang Y., Xu Y., Tan S., Zhang Y., Duan J., Yang Y. (2016). PiR-651 Promotes Tumor Formation in Non-Small Cell Lung Carcinoma through the Upregulation of Cyclin D1 and CDK4. Int. J. Mol. Med..

[144] Zhang S.J., Yao J., Shen B.Z., Li G.B., Kong S.S., Bi D.D., Pan S.H., Cheng B.L. (2018). Role of Piwi-Interacting RNA-651 in the Carcinogenesis of Non-Small Cell Lung Cancer. Oncol. Lett..

[145] Huang G., Hu H., Xue X., Shen S., Gao E., Guo G., Shen X., Zhang X. (2013). Altered Expression of PiRNAs and Their Relation with Clinicopathologic Features of Breast Cancer. Clin. Transl. Oncol..

[146] Hashim A., Rizzo F., Marchese G., Ravo M., Tarallo R., Nassa G., Giurato G., Santamaria G., Cordella A., Cantarella C. (2014). RNA Sequencing Identifies Specific PIWI-Interacting Small Noncoding RNA Expression Patterns in Breast Cancer. Oncotarget.

[147] Liang Y., Van Zant G. (2008). Aging Stem Cells, Latexin, and Longevity. Exp. Cell Res..

[148] Fu A., Jacobs D.I., Hoffman A.E., Zheng T., Zhu Y. (2015). PIWI-Interacting RNA 021285 Is Involved in Breast Tumorigenesis Possibly by Remodeling the Cancer Epigenome. Carcinogenesis.

[149] Lawson C.D., Fan C., Mitin N., Baker N.M., George S.D., Graham D.M., Perou C.M., Burridge K., Der C.J., Rossman K.L. (2016). Molecular and Cellular Pathobiology Rho GTPase Transcriptome Analysis Reveals Oncogenic Roles for Rho GTPase-Activating Proteins in Basal-like Breast Cancers. Cancer Res..

[150] Tan L., Mai D., Zhang B., Jiang X., Zhang J., Bai R., Ye Y., Li M., Pan L., Su J. (2019). PIWI-Interacting RNA-36712 Restrains Breast Cancer Progression and Chemoresistance by Interaction with SEPW1 Pseudogene SEPW1P RNA. Mol. Cancer.

[151] Hawkes W.C., Alkan Z. (2011). Delayed Cell Cycle Progression from SEPW1 Depletion Is P53- and P21-Dependent in MCF-7 Breast Cancer Cells. Biochem. Biophys. Res. Commun..

[152] Hawkes W.C., Printsev I., Alkan Z. (2012). Selenoprotein W Depletion Induces a P53- and P21-Dependent Delay in Cell Cycle Progression in RWPE-1 Prostate Epithelial Cells. J. Cell. Biochem..

[153] Wang S.P., Wang W.L., Chang Y.L., Wu C.T., Chao Y.C., Kao S.H., Yuan A., Lin C.W., Yang S.C., Chan W.K. (2009). P53 Controls Cancer Cell Invasion by Inducing the MDM2-Mediated Degradation of Slug. Nat. Cell Biol..

[154] Hajra K.M., Chen D.Y.S., Fearon E.R. (2002). The SLUG Zinc-Finger Protein Represses E-Cadherin in Breast Cancer. Cancer Res..

[155] He X., Chen X., Zhang X., Duan X., Pan T., Hu Q., Zhang Y., Zhong F., Liu J., Zhang H. (2015). An Lnc RNA (GAS5)/SnoRNA-Derived PiRNA Induces Activation of TRAIL Gene by Site-Specifically Recruiting MLL/COMPASS-like Complexes. Nucleic Acids Res..

[156] Zhang H., Ren Y., Xu H., Pang D., Duan C., Liu C. (2013). The Expression of Stem Cell Protein Piwil2 and PiR-932 in Breast Cancer. Surg. Oncol..

[157] Liang J., Wen J., Huang Z., Chen X., Zhang B., Chu L. (2019). Small Nucleolar RNAs: Insight Into Their Function in Cancer. Front. Oncol..

[158] Richard P., Darzacq X., Bertrand E., Jády B.E., Verheggen C., Kiss T. (2003). A Common Sequence Motif Determines the Cajal Body-Specific Localization of Box H/ACA ScaRNAs. EMBO J..

[159] Kiss A.M., Jády B.E., Bertrand E., Kiss T. (2004). Human Box H/ACA Pseudouridylation Guide RNA Machinery. Mol. Cell. Biol..

[160] Chi Y., Wang D., Wang J., Yu W., Yang J. (2019). Long Non-Coding RNA in the Pathogenesis of Cancers. Cells.

[161] Cavaillé J., Buiting K., Kiefmann M., Lalande M., Brannan C.I., Horsthemke B., Bachellerie J.P., Brosius J., Hüttenhofer A. (2000). Identification of Brain-Specific and Imprinted Small Nucleolar RNA Genes Exhibiting an Unusual Genomic Organization. Proc. Natl. Acad. Sci. USA.

[162] Kishore S., Khanna A., Zhang Z., Hui J., Balwierz P.J., Stefan M., Beach C., Nicholls R.D., Zavolan M., Stamm S. (2010). The SnoRNA MBII-52 (SNORD 115) Is Processed into Smaller RNAs and Regulates Alternative Splicing. Hum. Mol. Genet..

[163] Falaleeva M., Surface J., Shen M., de la Grange P., Stamm S. (2015). SNORD116 and SNORD115 Change Expression of Multiple Genes and Modify Each Other’s Activity. Gene.

[164] Michel C.I., Holley C.L., Scruggs B.S., Sidhu R., Brookheart R.T., Listenberger L.L., Behlke M.A., Ory D.S., Schaffer J.E. (2011). Small Nucleolar RNAs U32a, U33, and U35a Are Critical Mediators of Metabolic Stress. Cell Metab..

[165] Brandis K.A., Gale S., Jinn S., Langmade S.J., Dudley-Rucker N., Jiang H., Sidhu R., Ren A., Goldberg A., Schaffer J.E. (2013). Box C/D Small Nucleolar RNA (SnoRNA) U60 Regulates Intracellular Cholesterol Trafficking. J. Biol. Chem..

[166] Jinn S., Brandis K.A., Ren A., Chacko A., Dudley-Rucker N., Gale S.E., Sidhu R., Fujiwara H., Jiang H., Olsen B.N. (2015). SnoRNA U17 Regulates Cellular Cholesterol Trafficking. Cell Metab..

[167] Jorjani H., Kehr S., Jedlinski D.J., Gumienny R., Hertel J., Stadler P.F., Zavolan M., Gruber A.R. (2016). An Updated Human SnoRNAome. Nucleic Acids Res..

[168] Vogt P.K. (2001). PI 3-Kinase, MTOR, Protein Synthesis and Cancer. Trends Mol. Med..

[169] Boon K., Caron H.N., Van Asperen R., Valentijn L., Hermus M.C., Van Sluis P., Roobeek I., Weis I., Voûte P.A., Schwab M. (2001). N-Myc Enhances the Expression of a Large Set of Genes Functioning in Ribosome Biogenesis and Protein Synthesis. EMBO J..

[170] Su H., Xu T., Ganapathy S., Shadfan M., Long M., Huang T.H.M., Thompson I., Yuan Z.M. (2014). Elevated SnoRNA Biogenesis Is Essential in Breast Cancer. Oncogene.

[171] Watkins N.J., Lemm I., Ingelfinger D., Schneider C., Hoßbach M., Urlaub H., Lührmann R. (2004). Assembly and Maturation of the U3 SnoRNP in the Nucleoplasm in a Large Dynamic Multiprotein Complex. Mol. Cell.

[172] Yu F., Bracken C.P., Pillman K.A., Lawrence D.M., Goodall G.J., Callen D.F., Neilsen P.M. (2015). P53 Represses the Oncogenic Sno-MiR-28 Derived from a SnoRNA. PLoS ONE.

[173] Langhendries J.L., Nicolas E., Doumont G., Goldman S., Lafontaine D.L.J. (2016). The Human Box C/D SnoRNAs U3 and U8 Are Required for PrerRNA Processing and Tumorigenesis. Oncotarget.

[174] Gee H.E., Buffa F.M., Camps C., Ramachandran A., Leek R., Taylor M., Patil M., Sheldon H., Betts G., Homer J. (2011). The Small-Nucleolar RNAs Commonly Used for MicroRNA Normalisation Correlate with Tumour Pathology and Prognosis. Br. J. Cancer.

[175] Wang H., Tan Z., Hu H., Liu H., Wu T., Zheng C., Wang X., Luo Z., Wang J., Liu S. (2019). MicroRNA-21 Promotes Breast Cancer Proliferation and Metastasis by Targeting LZTFL1. BMC Cancer.

[176] Patterson D.G., Roberts J.T., King V.M., Houserova D., Barnhill E.C., Crucello A., Polska C.J., Brantley L.W., Kaufman G.C., Nguyen M. (2017). Human SnoRNA-93 Is Processed into a MicroRNA-like RNA That Promotes Breast Cancer Cell Invasion. Breast Cancer.

[177] Kim M.J., Jung W.H., Koo J.S. (2015). Expression of Sarcosine Metabolism-Related Proteins in Estrogen Receptor Negative Breast Cancer According to the Androgen Receptor and HER-2 Status. Int. J. Clin. Exp. Pathol..

[178] Watson C.N., Belli A., Di Pietro V. (2019). Small Non-Coding RNAs: New Class of Biomarkers and Potential Therapeutic Targets in Neurodegenerative Disease. Front. Genet..

[179] Galganski L., Urbanek M.O., Krzyzosiak W.J. (2017). Nuclear Speckles: Molecular Organization, Biological Function and Role in Disease. Nucleic Acids Res..

[180] Bohnsack M.T., Sloan K.E. (2018). Modifications in Small Nuclear RNAs and Their Roles in Spliceosome Assembly and Function. Biol. Chem..

[181] Padgett R.A. (2012). New Connections between Splicing and Human Disease. Trends Genet..

[182] Valadkhan S., Jaladat Y. (2010). The Spliceosomal Proteome: At the Heart of the Largest Cellular Ribonucleoprotein Machine. Proteomics.

[183] Wahl M.C., Will C.L., Lührmann R. (2009). The Spliceosome: Design Principles of a Dynamic RNP Machine. Cell.

[184] Cheng Z., Sun Y., Niu X., Shang Y., Ruan J., Chen Z., Gao S., Zhang T. (2017). Gene Expression Profiling Reveals U1 SnRNA Regulates Cancer Gene Expression. Oncotarget.

[185] Wang B.D., Lee N.H. (2018). Aberrant RNA Splicing in Cancer and Drug Resistance. Cancers.

[186] Dvinge H., Guenthoer J., Porter P.L., Bradley R.K. (2019). RNA Components of the Spliceosome Regulate Tissueand Cancer-Specific Alternative Splicing. Genome Res..

[187] Oh J.M., Venters C.C., Di C., Pinto A.M., Wan L., Younis I., Cai Z., Arai C., So B.R., Duan J. (2020). U1 SnRNP Regulates Cancer Cell Migration and Invasion in Vitro. Nat. Commun..

[188] Lee S.H., Singh I., Tisdale S., Abdel-Wahab O., Leslie C.S., Mayr C. (2018). Widespread Intronic Polyadenylation Inactivates Tumour Suppressor Genes in Leukaemia. Nature.

[189] Sandberg R., Neilson J.R., Sarma A., Sharp P.A., Burge C.B. (2008). Proliferating Cells Express MRNAs with Shortened 3′ Untranslated Regions and Fewer MicroRNA Target Sites. Science.

[190] Mayr C., Bartel D.P. (2009). Widespread Shortening of 3′UTRs by Alternative Cleavage and Polyadenylation Activates Oncogenes in Cancer Cells. Cell.

[191] Oh J.M., Di C., Venters C.C., Guo J., Arai C., So B.R., Pinto A.M., Zhang Z., Wan L., Younis I. (2017). U1 SnRNP Telescripting Regulates a Size-Function-Stratified Human Genome. Nat. Struct. Mol. Biol..

[192] Pardini B., Sabo A.A., Birolo G., Calin G.A. (2019). Noncoding RNAs in Extracellular Fluids as Cancer Biomarkers: The New Frontier of Liquid Biopsies. Cancers.

[193] Appaiah H.N., Goswami C.P., Mina L.A., Badve S., Sledge G.W., Liu Y., Nakshatri H. (2011). Persistent Upregulation of U6:SNORD44 Small RNA Ratio in the Serum of Breast Cancer Patients. Breast Cancer Res..

[194] Dana H., Chalbatani G.M., Mahmoodzadeh H., Karimloo R., Rezaiean O., Moradzadeh A., Mehmandoost N., Moazzen F., Mazraeh A., Marmari V. (2017). Molecular Mechanisms and Biological Functions of SiRNA. Int. J. Biomed. Sci..

[195] Endoh T., Ohtsuki T. (2009). Cellular SiRNA Delivery Using Cell-Penetrating Peptides Modified for Endosomal Escape. Adv. Drug Deliv. Rev..

[196] Chiu Y.L., Rana T.M. (2002). RNAi in Human Cells: Basic Structural and Functional Features of Small Interfering RNA. Mol. Cell.

[197] Levanova A., Poranen M.M. (2018). RNA Interference as a Prospective Tool for the Control of Human Viral Infections. Front. Microbiol..

[198] TenOever B.R. (2017). Questioning Antiviral RNAi in Mammals. Nat. Microbiol..

[199] Seo G.J., Kincaid R.P., Phanaksri T., Burke J.M., Pare J.M., Cox J.E., Hsiang T.Y., Krug R.M., Sullivan C.S. (2013). Reciprocal Inhibition between Intracellular Antiviral Signaling and the RNAi Machinery in Mammalian Cells. Cell Host Microbe.

[200] Bhat S.A., Ahmad S.M., Mumtaz P.T., Malik A.A., Dar M.A., Urwat U., Shah R.A., Ganai N.A. (2016). Long Non-Coding RNAs: Mechanism of Action and Functional Utility. Non Coding RNA Res..

[201] Tatiparti K., Sau S., Kashaw S.K., Iyer A.K. (2017). SiRNA Delivery Strategies: A Comprehensive Review of Recent Developments. Nanomaterials.

[202] Gao K., Huang L. (2009). Nonviral Methods for SiRNA Delivery. Mol. Pharm..

[203] Song E., Zhu P., Lee S.K., Chowdhury D., Kussman S., Dykxhoorn D.M., Feng Y., Palliser D., Weiner D.B., Shankar P. (2005). Antibody Mediated in Vivo Delivery of Small Interfering RNAs via Cell-Surface Receptors. Nat. Biotechnol..

[204] Dong Y., Siegwart D.J., Anderson D.G. (2019). Strategies, Design, and Chemistry in SiRNA Delivery Systems. Adv. Drug Deliv. Rev..

[205] Nishina K., Unno T., Uno Y., Kubodera T., Kanouchi T., Mizusawa H., Yokota T. (2008). Efficient in Vivo Delivery of SiRNA to the Liver by Conjugation of α-Tocopherol. Mol. Ther..

[206] Järve A., Muller J., Kim I.H., Rohr K., Maclean C., Fricker G., Massing U., Eberle F., Dalpke A., Fischer R. (2007). Surveillance of SiRNA Integrity by FRET Imaging. Nucleic Acids Res..

[207] Ohrt T., Mütze J., Staroske W., Weinmann L., Höck J., Crell K., Meister G., Schwille P. (2008). Fluorescence Correlation Spectroscopy and Fluorescence Cross-Correlation Spectroscopy Reveal the Cytoplasmic Origination of Loaded Nuclear RISC in Vivo in Human Cells. Nucleic Acids Res..

[208] Gagnon K.T., Li L., Chu Y., Janowski B.A., Corey D.R. (2014). RNAi Factors Are Present and Active in Human Cell Nuclei. Cell Rep..

[209] Rao D.D., Vorhies J.S., Senzer N., Nemunaitis J. (2009). SiRNA vs. ShRNA: Similarities and Differences. Adv. Drug Deliv. Rev..

[210] Wang W., Nag S.A., Zhang R. (2015). Targeting the NFκB Signaling Pathways for Breast Cancer Prevention and Therapy. Curr. Med. Chem..

[211] Yu H., Guo C., Feng B., Liu J., Chen X., Wang D., Teng L., Li Y., Yin Q., Zhang Z. (2016). Triple-Layered PH-Responsive Micelleplexes Loaded with SiRNA and Cisplatin Prodrug for NF-Kappa B Targeted Treatment of Metastatic Breast Cancer. Theranostics.

[212] Giridharan S., Srinivasan M. (2018). Mechanisms of NF-ΚB P65 and Strategies for Therapeutic Manipulation. J. Inflamm. Res..

[213] Koval A., Katanaev V.L. (2018). Dramatic Dysbalancing of the Wnt Pathway in Breast Cancers. Sci. Rep..

[214] Vaidya A.M., Sun Z., Ayat N., Schilb A., Liu X., Jiang H., Sun D., Scheidt J., Qian V., He S. (2019). Systemic Delivery of Tumor-Targeting SiRNA Nanoparticles against an Oncogenic LncRNA Facilitates Effective Triple-Negative Breast Cancer Therapy. Bioconjug. Chem..

[215] Liu Y., Tamimi R.M., Collins L.C., Schnitt S.J., Gilmore H.L., Connolly J.L., Colditz G.A. (2011). The Association between Vascular Endothelial Growth Factor Expression in Invasive Breast Cancer and Survival Varies with Intrinsic Subtypes and Use of Adjuvant Systemic Therapy: Results from the Nurses’ Health Study. Breast Cancer Res. Treat..

[216] Skobe M., Hawighorst T., Jackson D.G., Prevo R., Janes L., Velasco P., Riccardi L., Alitalo K., Claffey K., Detmar M. (2001). Induction of Tumor Lymphangiogenesis by VEGF-C Promotes Breast Cancer Metastasis. Nat. Med..

[217] Feng Q., Yu M.Z., Wang J.C., Hou W.J., Gao L.Y., Ma X.F., Pei X.W., Niu Y.J., Liu X.Y., Qiu C. (2014). Synergistic Inhibition of Breast Cancer by Co-Delivery of VEGF SiRNA and Paclitaxel via Vapreotide-Modified Core-Shell Nanoparticles. Biomaterials.

[218] Lee S.Y., Jang C., Lee K.A. (2014). Polo-Like Kinases (Plks), a Key Regulator of Cell Cycle and New Potential Target for Cancer Therapy. Dev. Reprod..

[219] Chabalier-Taste C., Brichese L., Racca C., Canitrot Y., Calsou P., Larminat F. (2016). Polo-like Kinase 1 Mediates BRCA1 Phosphorylation and Recruitment at DNA Double-Strand Breaks. Oncotarget.

[220] Ueda A., Oikawa K., Fujita K., Ishikawa A., Sato E., Ishikawa T., Kuroda M., Kanekura K. (2019). Therapeutic Potential of PLK1 Inhibition in Triple-Negative Breast Cancer. Lab. Investig..

[221] Morry J., Ngamcherdtrakul W., Gu S., Reda M., Castro D.J., Sangvanich T., Gray J.W., Yantasee W. (2017). Targeted Treatment of Metastatic Breast Cancer by PLK1 SiRNA Delivered by an Antioxidant Nanoparticle Platform. Mol. Cancer Ther..

[222] Nachreiner I., Hussain A., Wullner U., Machuy N., Meyer T., Fischer R., Gattenlöhner S., Meinhold-Heerlein I., Barth S., Tur M. (2019). Elimination of HER3-expressing Breast Cancer Cells Using Aptamer-siRNA Chimeras. Exp. Ther. Med..

[223] Zhang L., Mu C., Zhang T., Wang Y., Wang Y., Fan L., Liu C., Chen H., Shen J., Wei K. (2020). Systemic Delivery of Aptamer-Conjugated XBP1 SiRNA Nanoparticles for Efficient Suppression of HER2+ Breast Cancer. ACS Appl. Mater. Interfaces.

[224] Zhao L., Gu C., Gan Y., Shao L., Chen H., Zhu H. (2020). Exosome-Mediated SiRNA Delivery to Suppress Postoperative Breast Cancer Metastasis. J. Control. Release.

[225] Liu L., Qi L., Knifley T., Piecoro D.W., Rychahou P., Liu J., Mitov M.I., Martin J., Wang C., Wu J. (2019). S100A4 Alters Metabolism and Promotes Invasion of Lung Cancer Cells by Up-Regulating Mitochondrial Complex i Protein NDUFS2. J. Biol. Chem..

[226] Grum-Schwensen B., Klingelhöfer J., Beck M., Bonefeld M.M., Hamerlik P., Guldberg P., Grigorian M., Lukanidin E., Ambartsumian N. (2015). S100A4-Neutralizing Antibody Suppresses Spontaneous Tumor Progression, Pre-Metastatic Niche Formation and Alters T-Cell Polarization Balance. BMC Cancer.

[227] Curran M.A., Kim M., Montalvo W., Al-Shamkhani A., Allison J.P. (2011). Combination CTLA-4 Blockade and 4-1BB Activation Enhances Tumor Rejection by Increasing T-Cell Infiltration, Proliferation, and Cytokine Production. PLoS ONE.

[228] Gros A., Robbins P.F., Yao X., Li Y.F., Turcotte S., Tran E., Wunderlich J.R., Mixon A., Farid S., Dudley M.E. (2014). PD-1 Identifies the Patient-Specific CD8+ Tumor-Reactive Repertoire Infiltrating Human Tumors. J. Clin. Investig..

[229] Rosenberg S.A., Restifo N.P., Yang J.C., Morgan R.A., Dudley M.E. (2008). Adoptive Cell Transfer: A Clinical Path to Effective Cancer Immunotherapy. Nat. Rev. Cancer.

[230] Muenst S., Schaerli A.R., Gao F., Däster S., Trella E., Droeser R.A., Muraro M.G., Zajac P., Zanetti R., Gillanders W.E. (2014). Expression of Programmed Death Ligand 1 (PD-L1) Is Associated with Poor Prognosis in Human Breast Cancer. Breast Cancer Res. Treat..

[231] Wu Y., Gu W., Li J., Chen C., Xu Z.P. (2019). Silencing PD-1 and PD-L1 with Nanoparticle-Delivered Small Interfering RNA Increases Cytotoxicity of Tumor-Infiltrating Lymphocytes. Nanomedicine.

